# The TGFβ-signaling pathway and colorectal cancer: associations between dysregulated genes and miRNAs

**DOI:** 10.1186/s12967-018-1566-8

**Published:** 2018-07-09

**Authors:** Andrew J. Pellatt, Lila E. Mullany, Jennifer S. Herrick, Lori C. Sakoda, Roger K. Wolff, Wade S. Samowitz, Martha L. Slattery

**Affiliations:** 1 School of Medicine, Tulane University, New Orleans, LA USA; 2 Department of Medicine, University of Utah, 383 Colorow, Salt Lake City, UT 84108 USA; 3 Division of Research, Kaiser Permanente Northern California, Oakland, CA USA; 4 Department of Pathology, University of Utah, Salt Lake City, UT USA

**Keywords:** TGFβ, Colorectal cancer, miRNA, mRNA

## Abstract

**Background:**

The TGFβ-signaling pathway plays an important role in the pathogenesis of colorectal cancer (CRC). Loss of function of several genes within this pathway, such as bone morphogenetic proteins (BMPs) have been seen as key events in CRC progression.

**Methods:**

In this study we comprehensively evaluate differential gene expression (RNASeq) of 81 genes in the TGFβ-signaling pathway and evaluate how dysregulated genes are associated with miRNA expression (Agilent Human miRNA Microarray V19.0). We utilize paired carcinoma and normal tissue from 217 CRC cases. We evaluate the associations between differentially expressed genes and miRNAs and sex, age, disease stage, and survival months.

**Results:**

Thirteen genes were significantly downregulated and 14 were significantly upregulated after considering fold change (FC) of > 1.50 or < 0.67 and multiple comparison adjustment. Bone morphogenetic protein genes *BMP5*, *BMP6*, and *BMP2* and growth differentiation factor *GDF7* were downregulated. *BMP4*, *BMP7*, *INHBA* (Inhibin beta A), *TGFBR1*, *TGFB2*, *TGIF1*, *TGIF2*, and *TFDP1* were upregulated. In general, genes with the greatest dysregulation, such as *BMP5* (FC 0.17, *BMP6* (FC 0.25), *BMP2* (FC 0.32), *CDKN2B* (FC 0.32), *MYC* (FC 3.70), *BMP7* (FC 4.17), and *INHBA* (FC 9.34) showed dysregulation in the majority of the population (84.3, 77.4, 81.1, 80.2, 82.0, 51.2, and 75.1% respectively). Four genes, *TGFBR2*, *ID4*, *ID1*, and *PITX2*, were un-associated or slightly upregulated in microsatellite-stable (MSS) tumors while downregulated in microsatellite-unstable (MSI) tumors. Eight dysregulated genes were associated with miRNA differential expression. *E2F5* and *THBS1* were associated with one or two miRNAs; *RBL1*, *TGFBR1*, *TGIF2*, and *INHBA* were associated with seven or more miRNAs with multiple seed-region matches. Evaluation of the joint effects of mRNA:miRNA identified interactions that were stronger in more advanced disease stages and varied by survival months.

**Conclusion:**

These data support an interaction between miRNAs and genes in the TGFβ-signaling pathway in association with CRC risk. These interactions are associated with unique clinical characteristics that may provide targets for further investigations.

**Electronic supplementary material:**

The online version of this article (10.1186/s12967-018-1566-8) contains supplementary material, which is available to authorized users.

## Background

The TGFβ-signaling pathway is important in the tumorigenesis of colorectal cancer (CRC) [1]. This pathway is a regulator of cellular proliferation, differentiation, apoptosis, and extracellular matrix remodeling, and also is involved in angiogenesis and inflammation [2, 3]. It has been previously reported that components of the TGFβ-signaling pathway are mutated in 27% of non-hypermutated tumors and 87% of hypermutated tumors [4], and that inactivation of the pathway is a common event in CRC tumorigenesis [4]. The TGFβ family of cytokine genes includes three isoforms of TGF-β, TGF-β1, TGF-β2, and TGF-β3, the type I receptors (TβR1 and ALK1) and a type II receptor (TβRII) [5]. Other components of the TGFβ-signaling pathway include SMAD genes, key intracellular mediators of the transcriptional responses to TGF-β [6] and bone morphogenetic proteins (BMP), which trigger a SMAD-signaling cascade that is linked to cell proliferation and cellular growth [7, 8]. Growth differentiation factors (GDF) [9] and their receptors are components of the TGFβ superfamily as are Activin/inhibin and their receptors [10]. BMP ligands bind to type 1 [BMPR1A, BMPR1B, Activin A receptor type 1 (ACVR1), and Activin receptor-like kinase 1 (ACVRL1)] and type 2 receptors [BMPR2, Activin A receptor type IIA (ACVR2A) and type IIB (ACVR2B)] [9]. While both type 1 and 2 receptors are needed for BMP signaling, type I receptors bind with a higher affinity than type II receptors. *BMPR1A* has been reported as being inactivated in some studies focusing on familial syndromes such as mixed polyposis syndrome and familial juvenile polyposis [11–13]. Other factors that regulate TGFβ and its receptors are BAMBI (BMP and activing membrane bound inhibitor) [14], THBS1 (thrombospondin 1 also known as TSP1) [15], LEFTY (left–right determination factor, Factor 2 also known as TGFβ4) [16], and FST (Follistatin) [17].

It has been hypothesized that miRNAs, small, non-protein-coding RNA molecules involved in the regulation of gene expression either by post-transcriptionally suppressing mRNA translation or by causing mRNA degradation [18–23], may work with the TGFβ-signaling pathway to mediate cell growth and promote tumorigenesis [24]. MiRNAs have been linked to TGFβ-signaling pathway in a variety of diseases, usually in studies which only examined few miRNAs and genes within the signaling pathway. For instance, miR-181a has been shown to have its expression altered by TGFβ [24, 25], miR-494 and miR-126-5p have been linked to *BMP4* in the regulation of angiogenesis [26]; miR-98 with *TBSP1* expression in asthma [27]; miR-590-5p with downregulation of TGFβ signaling in cardiosphere-derived stem cells [28]; the miR-17-92 cluster with cell proliferation in mouse mesenchymal cells [29]; and miR-140-5p with tumor growth [30]. MiRNAs have also been linked with various disease processes in CRC. For example, miR-200a-3p regulates epithelial mesenchymal transition in CRC [31]; and miR-21-5p is upregulated in CRC, while its inhibition can hinder CRC tumor growth [31]. Although the TGFβ-signaling pathway appears to be important for CRC, few studies have looked at this pathway with miRNAs. We have previously examined miRNA expression with SNPs in 21 genes associated with the TGFβ-signaling pathway and found that expression of several miRNAs varied by SNPs in *TGFβ1* in normal mucosa [32]. Additionally, other groups have found that specific miRNAs play a significant role in CRC via the TGFβ pathway. MiR-193b has been shown to play an important role in CRC by promoting cellular proliferation via SMAD3 [33]; miRs-203, 181d and 182 regulate genes, including TGF-β2 and RUNX2, involved in CRC proliferation, differentiation, and invasion [34]; and miR-34a mediates oxaliplatin resistance in CRC via the TGFβ/SMAD4 pathway [35].

In this study, we comprehensively examined expression differences between carcinoma and normal mucosa with all genes identified in the TGFβ-signaling pathway. For those genes that had significant differences in expression when considering level of expression (> 1.50 fold change (FC) or < 0.67 FC), we assessed their association with expression levels of over 800 miRNAs commonly expressed in CRC. We evaluated associations for overall CRC as well as for specific tumor molecular phenotype, i.e. tumors with or without mismatch repair deficiency, and how these associations relate to clinical features such as disease stage and survival months. Our goal is to obtain a better understanding of how miRNAs relate to the TGFβ-signaling pathway in CRC carcinogenesis. We believe that our epidemiological approach, using population-based cases, is an initial step in identifying associations that can be further examined in targeted laboratory studies with the goal of identification of targeted therapeutics.

## Methods

### Study participants

Participants come from two population-based case–control studies that included all incident colon and rectal cancer diagnosed between 30 and 79 years of age in Utah or at Kaiser Permanente in Northern California (KPNC). Diagnosis was verified by tumor registry data as a first primary adenocarcinoma of the colon or rectum with a diagnosis date between October 1991 and September 1994 (colon study) and between May 1997 and May 2001 (rectal study) [36]. Participants were non-Hispanic white, Hispanic, or black for the colon cancer study; the rectal cancer study also included people who self-reported being Asian [37, 38]. The Institutional Review Boards at the University of Utah and at KPNC approved the study.

### Ethics, consent, and permissions

Study participants signed informed consent. The Institutional Review Boards at the University of Utah and at KPNC approved this study. Individual-level participant data are not reported.

### RNA processing

Formalin-fixed paraffin embedded tissue from either surgery or the initial biopsy was used to extract RNA. RNA was extracted, isolated and purified from carcinoma tissue and adjacent normal mucosa as previously described [39]. Differences in RNA quality were not observed based on age of the tissue; all samples were of a high quality.

### mRNA: RNA-Seq Sequencing Library Preparation and Data Processing

RNA from 245 colorectal carcinoma and normal mucosa pairs was chosen for sequencing based on availability of RNA and high quality miRNA data; 217 pairs passed quality control (QC) and were used in these analyses. RNA library construction was done with the Illumina TruSeq Stranded Total RNA Sample Preparation Kit with Ribo-Zero. The samples were then fragmented and primed for cDNA synthesis, adapters were then ligated onto the cDNA, and the resulting samples were then amplified using PCR; the amplified library was then purified using Agencount AMPure XP beads. A more detailed description of the methods can be found in our previous work [40]. Illumina TruSeq v3 single read flow cell and a 50 cycle single-read sequence run was performed on an Illumina HiSeq instrument. Reads were aligned to a sequence database containing the human genome (build GRCh37/hg19, February 2009 from genome.ucsc.edu) and alignment was performed using novoalign v2.08.01. Total gene counts were calculated for each exon and UTR of the genes using a list of gene coordinates obtained from http://genome.ucsc.edu. We disregarded genes that were not expressed in our RNA-Seq data or for which the expression was missing for the majority of samples [40].

### miRNA expression

The Agilent Human miRNA Microarray V19.0 was used. Data were required to pass stringent QC parameters established by Agilent that included tests for excessive background fluorescence, excessive variation among probe sequence replicates on the array, and measures of the total gene signal on the array to assess low signal. Samples failing to meet quality standards were re-labeled, hybridized to arrays, and re-scanned. If a sample failed QC assessment a second time, the sample was excluded from analysis. The repeatability associated with this microarray was extremely high (r = 0.98) [36]; comparison of miRNA expression levels obtained from the Agilent microarray to those obtained from qPCR had an agreement of 100% in terms of directionality of findings and the FCs were almost identical [41]. To normalize differences in miRNA expression that could be attributed to the array, amount of RNA, location on array, or factors that could erroneously influence miRNA expression levels, total gene signal was normalized by multiplying each sample by a scaling factor which was the median of the 75th percentile of all the samples divided by the individual 75th percentile of each sample [42].

### TGFβ-signaling pathway genes

The Kyoto encyclopedia of genes and genomes (KEGG) (www.genome.jp/kegg-bin/show_pathway?hsa04350) pathway map for TGFβ-signaling was used to identify genes within this pathway. Using this pathway map, we identified 84 genes, 81 of which had sufficient expression in CRC tissue for inclusion in the study (Additional file 1: Table S1).

### Statistical methods

We utilized a negative binomial mixed effects model in SAS (accounting for paired carcinoma/normal status) to determine genes in the TGFβ-signaling pathway that had a significant difference in expression between individually paired colorectal carcinoma and normal mucosa (i.e. differentially expressed). In this test we offset the overall exposure as the log expression of all identified protein-coding genes (n = 17,461). The Benjamini and Hochberg [43] procedure was used to control the false discovery rate (FDR) using a value of 0.05 or less. We calculated FC using the population means to give an estimate of the magnitude of difference in expression to identify mRNA and miRNAs. Population means were used because in some instances there was no expression in either the tumor or normal, making individual FCs impossible to calculate. Utilization of the population means we believe gave the best estimate of FC in the population. We calculated level of expression of each gene by dividing the total expression for that gene in an individual by the total expression of all protein-coding genes per million transcripts (RPMPCG or reads per million protein-coding genes). We focused on those genes with FC of > 1.50 or < 0.67 for analysis with differential miRNA expression since these levels of FC may have a greater biological significance than FCs closer to one. A FC of greater than one indicates a positive differential expression (i.e. up-regulated in carcinoma), while a FC between zero and one indicates a negative differential expression (i.e. down-regulated in carcinoma). We also report at the population level the percentage of the population with FC > 1.50 or < 0.67 to provide an estimate of the prevalence of these changes in the population.

We evaluated clinical indicators of age, sex, AJCC disease stage, and survival months with those genes that were dysregulated using Spearman correlation coefficients. Additionally we evaluated miRNA and mRNA associations for specific categories of sex, age (< 55, 55–65, and > 65), AJCC disease stage, and survival months (24, 24–60, and > 60 months).

There were 814 miRNAs expressed in greater than 20% of normal colorectal mucosa that were analyzed; differential expression was calculated using subject-level paired data as the expression in the carcinoma tissue minus the expression in the normal mucosa. In these analyses, we fit a least squares linear regression model to the RPMPCG differential expression levels and miRNA differential expression levels. P-values were generated using the bootstrap method by creating a distribution of 10,000 F statistics derived by resampling the residuals from the null hypothesis model of no association between gene expression and miRNA expression using the boot package in R. Linear models were adjusted for age and sex. Multiplicity adjustments for gene/miRNA associations were made at the gene level using the FDR by Benjamini and Hochberg [43].

### Bioinformatics analysis

We analyzed miRNAs and targeted mRNAs for seed region matches. The mRNA 3′ UTR FASTA as well as the seed region sequence of the associated miRNA were analyzed to determine seed region pairings between miRNA and mRNA. MiRNA seed regions were calculated as described in our previous work [44]; we calculated and included seeds of six, seven, and eight nucleotides in length. Our hypothesis is that a seed-region match would increase the likelihood that identified genes associated with a specific miRNA were more likely to have a direct association (as indicated by a negative beta coefficient) given a higher propensity for binding and thus mRNA degradation. We used mRNA FASTA sequences generated from both GRCh37 and GRCh38 Homo sapiens alignments, using UCSC Table Browser (https://genome.ucsc.edu/cgi-bin/hgTables) [45]. We downloaded FASTA sequences that matched our Ensembl IDs and had a consensus coding sequences available. Analyses were done using scripts in R 3.2.3 and in perl 5.018002.

### Data accessibility

Utah miRNA data are available through GEO (https://www.ncbi.nlm.nih.gov/geo/query/acc.cgi?acc=GSE115513). Other data will be shared in accord with the signed consent and approved IRB studies. Contact study authors for data access.

## Results

Colon cancer cases comprised 77.9% of the study population (Table 1). The majority of cases were male (54.4%), non-Hispanic white (74.2%) and were diagnosed with a microsatellite stable (MSS) tumor. The mean age at diagnosis was 64.8 years.

**Table 1:** Description of study population

	N	%
Site		
Colon	169	77.9
Rectal	48	22.1
Sex		
Male	118	54.4
Female	99	45.6
Age		
Mean (SD)	64.8 (10.1)	
Race		
Non-Hispanic White	161	74.2
Hispanic	14	6.5
Non-Hispanic Black	8	3.7
Unknown	34	15.7
Tumor phenotype		
MSS	187	86.60
MSI	29	13.40

### Gene expression results

**Fig. 1:**
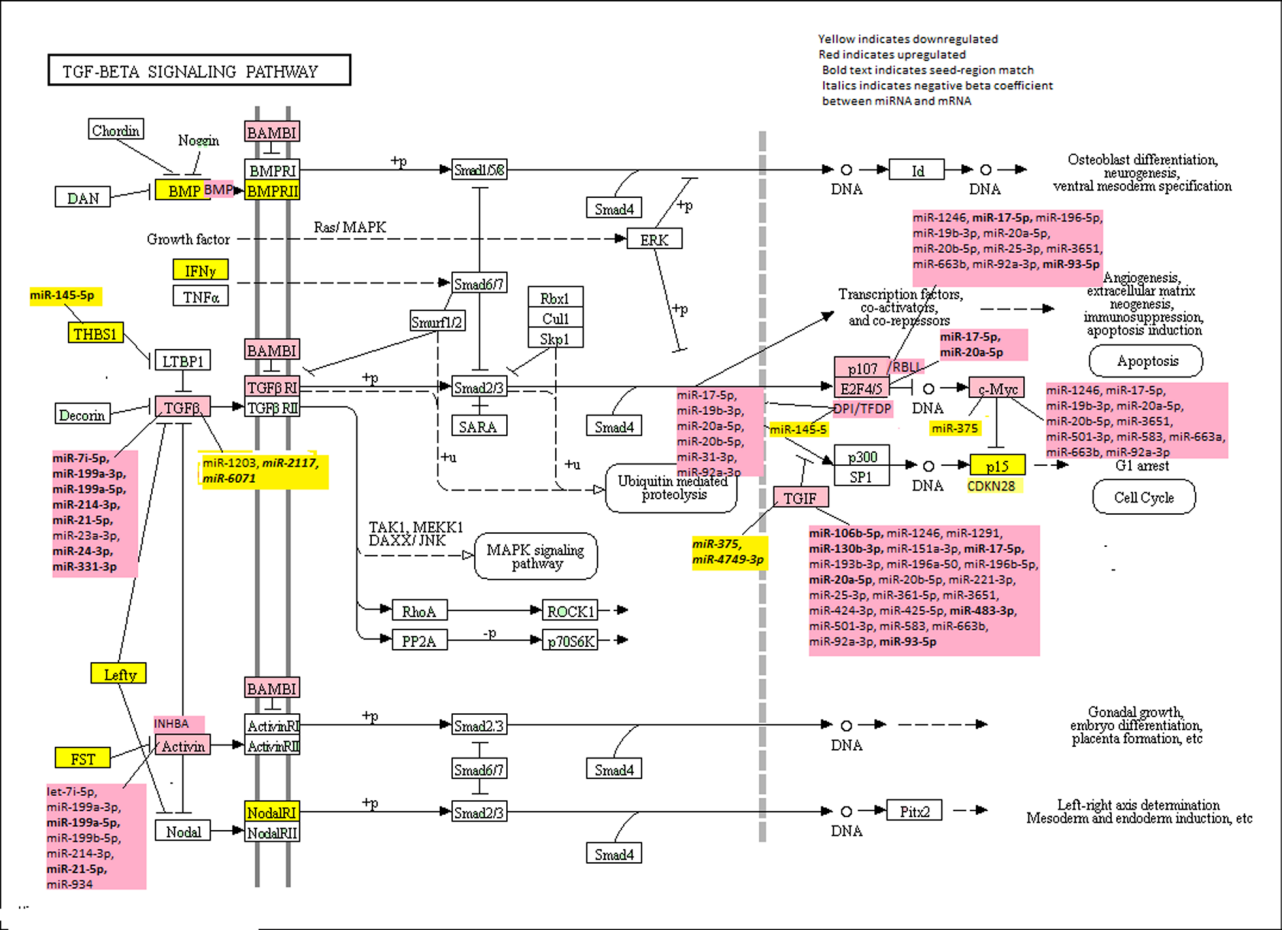
Dysregulated genes and associated miRNAs in the KEGG TGFβ-signaling pathway

Of the 81 genes analyzed, 27 showed statistically significant differences in expression between carcinoma and normal mucosa with a more meaningful FC when tumor sub-site was not considered (Table 2). Of these 27 genes, 13 were downregulated and 14 were up-regulated. Of the downregulated genes, three were part of the BMP family (*BMP5, BMP6*, and *BMP2*) and two were part of the growth differentiation factor family (*GDF7* and *GDF6*). *INHBA* (Inhibin beta A), which encodes for a subunit of activin and inhibin, was strongly upregulated, while the gene encoding activin type 1 receptor, *ACVR1C*, was downregulated. Two BMPs (*BMP4* and *BMP7*) were upregulated. *TGFBR1, TGFB2, TGIF1, TGIF2*, and *TFDP1* were upregulated. Other downregulated genes were: *IFNG* and *CDKN2B* (FC 0.32), *AMHR2* and *LEFTY2* (FC 0.43), *LEFTY1* (FC 0.51), *FST* (FC 0.59), and *THBS1* (FC 0.66). Other upregulated genes were: *E2F5* (FC 1.52), *AMH* (FC 1.74), *BAMBI* (FC 1.85), *INHBB* (FC) 1.91), *RBL1* (FC 2.30), and *MYC* (FC 3.70). While not all individuals had upregulated or downregulated expression in expression, when considering the entire population these genes were significantly up or downregulated. The stronger the FC in terms of being downregulated or upregulated the greater likelihood that more individuals in the population had that tumor characteristic. However, there are exceptions to this. For instance, *LEFTY2* and *AMHR2* both had an overall FC of 0.43, yet only 34.1 and 18.4% of individuals in the population had a FC of < 0.67 for these genes respectively. This illustrates the variability of gene expression in the population, but also suggest that some genes such as *BMP5 BMP6*, and *BMP2* may be better markers of CRC given that 84.3, 77.4, and 81.1% of the population have significant downregulated expression. Figure 1 shows the KEGG TGFβ-Signaling Pathway and those genes that are up and downregulated within the pathway.

**Table 2:** Differentially gene expression in the TGFβ-signaling pathway

Gene name	Tumor mean	Tumor SD	Normal mean	Normal SD	Fold change	(95% CI)	Adjusted *P* value	% FC < 0.67	% FC > 1.5
*BMP5*	3.93	0.30	22.50	1.61	0.17	(0.14, 0.21)	3.54E−39	84.3	3.7
*BMP6*	4.29	0.31	17.05	1.06	0.25	(0.21, 0.30)	2.55E−35	77.4	4.1
*IFNG*	0.78	0.12	2.48	0.31	0.32	(0.22, 0.45)	2.10E−09	47.0	3.7
*BMP2*	20.73	1.04	64.78	2.92	0.32	(0.28, 0.36)	2.85E−41	81.1	4.1
*CDKN2B*	24.21	1.23	74.65	3.43	0.32	(0.29, 0.37)	1.57E−41	80.2	6.0
*GDF7*	1.48	0.14	3.63	0.30	0.41	(0.32, 0.51)	9.55E−13	55.3	7.8
*AMHR2*	0.16	0.05	0.38	0.12	0.43	(0.24, 0.78)	7.72E−03	18.4	1.8
*LEFTY2*	0.68	0.14	1.57	0.32	0.43	(0.31, 0.60)	2.11E−06	34.1	7.4
*ACVR1C*	7.52	0.45	14.84	0.89	0.51	(0.43, 0.60)	2.99E−13	64.1	10.6
*LEFTY1*	32.87	3.41	64.73	5.42	0.51	(0.40, 0.65)	1.84E−07	61.3	15.7
*FST*	1.87	0.20	3.18	0.36	0.59	(0.45, 0.76)	1.21E−04	37.8	6.9
*GDF6*	0.86	0.12	1.46	0.22	0.59	(0.42, 0.83)	3.37E−03	30.4	6.5
*THBS1*	585.51	23.55	881.12	35.29	0.66	(0.61, 0.72)	1.76E−17	50.7	10.1
*MAPK3*	62.34	2.09	92.73	3.04	0.67	(0.62, 0.73)	4.62E−18	50.7	6.9
*SMAD1*	27.03	0.89	36.99	1.22	0.73	(0.67, 0.80)	1.75E−11	46.1	9.2
*CHRD*	6.54	0.44	8.90	0.59	0.73	(0.62, 0.87)	4.89E−04	41.9	15.7
*SMAD4*	89.84	2.47	119.48	3.27	0.75	(0.71, 0.80)	3.59E−16	37.8	5.5
*SMAD7*	31.79	1.14	40.98	1.46	0.78	(0.70, 0.85)	1.05E−06	41.0	15.7
*SMAD9*	26.96	1.61	34.30	1.95	0.79	(0.68, 0.91)	1.62E−03	49.3	19.8
*ZFYVE9*	50.10	1.37	62.66	1.71	0.80	(0.74, 0.86)	2.93E−08	38.7	7.4
*ID3*	31.30	1.51	38.32	1.79	0.82	(0.73, 0.91)	6.80E−04	43.3	18.4
*SMAD2*	161.37	4.37	194.68	5.24	0.83	(0.78, 0.88)	4.56E−09	33.6	8.8
*ACVR1B*	94.45	2.49	112.99	2.93	0.84	(0.78, 0.90)	1.88E−06	35.9	10.1
*NBL1*	181.16	5.41	213.28	6.34	0.85	(0.80, 0.91)	4.56E−06	33.6	10.6
*SMAD3*	106.05	2.59	124.63	3.00	0.85	(0.80, 0.91)	2.27E−06	28.1	9.2
*PPP2CB*	55.72	1.63	65.30	1.88	0.85	(0.79, 0.92)	8.37E−05	35.5	12.4
*EP300*	293.90	3.99	339.29	4.67	0.87	(0.84, 0.90)	8.90E−14	16.6	3.2
*RPS6KB2*	35.48	0.90	40.51	1.06	0.88	(0.82, 0.93)	1.16E−04	28.6	14.3
*ACVR2A*	39.04	1.43	43.73	1.60	0.89	(0.82, 0.98)	1.70E−02	28.6	16.6
*RBX1*	20.82	0.70	23.31	0.83	0.89	(0.82, 0.97)	1.35E−02	29.0	17.5
*BMP8B*	21.66	0.75	24.25	0.87	0.89	(0.81, 0.98)	2.30E−02	35.0	20.7
*NODAL*	2.36	0.20	2.63	0.23	0.90	(0.71, 1.14)	4.10E−01	35.0	11.5
*INHBE*	0.80	0.10	0.86	0.12	0.93	(0.67, 1.30)	7.08E−01	28.1	5.5
*TNF*	1.95	0.20	2.04	0.25	0.96	(0.72, 1.27)	7.71E−01	26.3	10.1
*CREBBP*	264.23	3.97	273.44	4.17	0.97	(0.93, 1.00)	7.19E−02	8.3	5.5
*ROCK1*	143.22	3.87	148.19	4.03	0.97	(0.91, 1.02)	2.61E−01	19.4	15.2
*DCN*	73.70	4.09	75.88	3.96	0.97	(0.84, 1.12)	7.08E−01	38.2	28.1
*GDF5*	0.19	0.06	0.20	0.06	0.99	(0.58, 1.70)	9.72E−01	14.7	3.2
*ZFYVE16*	102.11	2.67	101.55	2.72	1.01	(0.95, 1.07)	8.66E−01	15.2	19.4
*MAPK1*	183.50	3.04	179.79	3.01	1.02	(0.98, 1.07)	3.91E−01	6.5	10.1
*TGFB1*	41.60	1.76	39.64	1.64	1.05	(0.94, 1.17)	3.99E−01	33.2	29.0
*SMURF1*	112.79	2.32	106.62	2.22	1.06	(1.00, 1.12)	4.85E−02	11.1	20.7
*PPP2R1B*	81.03	1.94	76.22	1.88	1.06	(1.00, 1.13)	5.57E−02	11.1	16.6
*SP1*	287.39	4.77	266.86	4.51	1.08	(1.03, 1.12)	4.71E−04	5.5	10.6
*ID2*	26.59	1.11	24.40	1.03	1.09	(0.98, 1.22)	1.41E−01	25.8	31.8
*TGFBR2*	197.09	4.95	178.93	4.49	1.10	(1.03, 1.18)	7.47E−03	18.9	28.6
*PPP2R1A*	142.03	2.87	127.10	2.65	1.12	(1.07, 1.17)	1.18E−05	8.3	18.9
*BMPR1A*	20.81	0.79	18.58	0.74	1.12	(1.01, 1.24)	3.21E−02	21.2	26.7
*SKP1*	91.51	2.16	81.23	1.97	1.13	(1.06, 1.19)	1.28E−04	12.0	20.3
*PPP2CA*	101.95	2.74	90.28	2.47	1.13	(1.06, 1.20)	1.85E−04	10.6	21.7
*BMPR1B*	3.21	0.39	2.83	0.35	1.13	(0.85, 1.51)	4.18E−01	27.6	11.5
*TGFB3*	16.83	0.94	14.40	0.78	1.17	(1.01, 1.35)	4.71E−02	31.3	31.3
*ACVR1*	33.10	0.98	28.22	0.88	1.17	(1.08, 1.28)	4.18E−04	15.7	36.4
*ID4*	13.08	0.76	11.09	0.67	1.18	(1.00, 1.39)	6.14E−02	31.8	32.7
*ID1*	53.99	3.59	45.77	2.69	1.18	(1.00, 1.39)	5.57E−02	37.3	33.2
*PITX2*	11.58	1.16	9.64	0.99	1.20	(0.90, 1.59)	2.34E−01	23.0	26.7
*RPS6KB1*	49.77	1.39	41.07	1.18	1.21	(1.13, 1.30)	4.70E−07	8.8	27.6
*BMP8A*	5.36	0.39	4.37	0.33	1.23	(1.03, 1.46)	2.56E−02	28.6	25.3
*E2F4*	71.86	1.84	58.20	1.54	1.23	(1.17, 1.31)	1.97E−11	10.6	32.3
*ACVR2B*	14.96	0.59	12.09	0.52	1.24	(1.12, 1.36)	3.44E−05	20.3	36.9
*BMPR2*	219.08	5.39	176.91	4.38	1.24	(1.17, 1.32)	1.08E−10	10.6	30.4
*LTBP1*	77.75	2.98	61.80	2.43	1.26	(1.13, 1.40)	7.76E−05	23.0	36.9
*SMAD6*	21.19	0.93	16.42	0.78	1.29	(1.15, 1.44)	2.26E−05	18.4	39.6
*SMAD5*	124.87	3.71	94.20	2.84	1.33	(1.24, 1.41)	3.30E−15	8.8	35.5
*SMURF2*	51.22	1.61	38.21	1.27	1.34	(1.24, 1.45)	1.04E−11	11.1	39.6
*RHOA*	282.98	5.14	209.50	3.91	1.35	(1.29, 1.42)	4.34E−26	3.7	39.6
*CUL1*	74.71	1.95	53.22	1.45	1.40	(1.31, 1.50)	9.04E−19	6.5	40.1
*E2F5*	46.80	1.65	30.87	1.18	1.52	(1.38, 1.67)	3.20E−15	11.5	48.4
*TGFBR1*	106.44	3.40	70.13	2.27	1.52	(1.40, 1.65)	3.31E−18	6.5	50.7
*TGIF1*	81.12	2.46	50.35	1.61	1.61	(1.49, 1.75)	7.21E−24	8.3	55.3
*AMH*	5.92	0.63	3.39	0.36	1.74	(1.37, 2.22)	1.63E−05	27.6	28.1
*BAMBI*	9.61	0.80	5.20	0.46	1.85	(1.48, 2.30)	2.72E−07	25.8	32.3
*INHBB*	5.87	0.55	3.08	0.30	1.91	(1.52, 2.39)	1.43E−07	22.1	30.4
*TGFB2*	9.23	0.60	4.52	0.34	2.04	(1.71, 2.44)	2.96E−13	16.1	37.8
*BMP4*	48.63	2.94	22.37	1.33	2.17	(1.88, 2.52)	6.72E−20	13.8	53.0
*TFDP1*	119.20	3.35	54.04	1.61	2.21	(2.03, 2.39)	1.55E−47	3.2	76.5
*RBL1*	53.52	1.98	23.32	0.93	2.30	(2.08, 2.54)	4.37E−39	7.8	69.6
*TGIF2*	58.87	2.18	22.72	0.91	2.59	(2.33, 2.88)	5.45E−42	6.5	71.0
*MYC*	181.11	8.63	49.00	2.42	3.70	(3.28, 4.17)	2.33E−53	6.0	82.0
*BMP7*	37.91	3.18	9.09	0.80	4.17	(3.34, 5.20)	4.84E−27	16.6	51.2
*INHBA*	125.48	8.60	13.43	1.08	9.34	(7.67, 11.39)	5.94E−56	4.6	75.1

A small number of genes had either slightly stronger associations (as indicated by FC) for the MSS (Additional file 1: Table S2) or MSI (Additional file 1: Table S3) phenotype. Associations were most similar for MSS tumors and overall tumors; one gene (*THBS1*) was associated overall (FC 0.66) and for MSS-specific tumors (FC 0.68) that would not have merited inclusion by our cutpoints for FC. *AMH* (anti-Mullerian Hormone) was upregulated in overall tumors (FC 1.74) to a greater effect than for MSS tumors (FC 1.48). There were considerably more differences observed between MSI tumors and overall tumors. Seven genes were downregulated in MSI tumors with a much greater effect than for MSS tumors. Two of these genes were much more strongly downregulated in MSI tumors (*ID3* FC_MSS_ 0.86 and FC_MSI_I 0.52; *THBS1* FC_MSS_ 0.68 and FC_MSI_ 0.58). The other five genes were slightly upregulated in MSS tumors while downregulated in MSI tumors (*INHBE* FC_MSS_ 1.06 and FC_MSI_ 0.52; *TGFBR2* FC_MSS_ 1.18 and FC_MSI_ 0.61; *ID4* FC_MSS_ 1.26 and FC_MSI_ 0.62; *ID1* FC_MSS_ 1.30 and FC_MSI_ 0.38; *PITX2* FC_MSS_ 2.36 and FC_MSI_ 0.52). *AMH* was much more strongly upregulated for MSI tumors (FC 3.41).

For those genes that had significant differential expression with a FC of < 0.67 or > 1.50 we further evaluated their associations with sex, age, disease stage and survival months (Table 3). There were few correlations with any of these factors that we consider strong, although correlations > 0.134 were statistically significant with p values < 0.05. The strongest correlations for these factors were: *INHBB* with sex (r = − 0.175) indicating slightly stronger *CDKN2B* expression levels among women; *BMP2* with age (r = 0.224) indicating less expression with increasing age; *CDKN2B* with disease stage (r = 0.197) indicating greater correlation with more advanced disease stage; and *BMP4* with survival (r = − 0.174) indicating worse survival with greater BMP4 expression.

**Table 3:** Spearman correlations between significant differentially expressed mRNA and clinical features

Gene	Sex		Age		Disease stage		Survival months	
	(N = 217)	p-value	(N = 217)	p-value	(N = 214)	p-value	(N = 216)	p-value
*RBL1*	− 0.013	0.85	− 0.134	0.049	− 0.108	0.11	0.100	0.14
*TGFB2*	− 0.081	0.23	− 0.089	0.19	0.114	0.09	− 0.122	0.07
*BAMBI*	− 0.073	0.28	0.055	0.42	0.037	0.59	− 0.107	0.12
*BMP7*	− 0.080	0.24	− 0.152	0.03	0.085	0.22	− 0.131	0.05
*AMH*	− 0.058	0.39	0.092	0.18	− 0.006	0.93	0.008	0.91
*TGFBR1*	− 0.027	0.69	− 0.060	0.38	− 0.011	0.87	0.044	0.52
*IFNG*	0.084	0.22	0.087	0.20	− 0.089	0.20	0.081	0.24
*BMP5*	− 0.053	0.44	0.007	0.91	− 0.010	0.88	0.095	0.16
*SMAD5*	− 0.074	0.28	− 0.052	0.44	0.035	0.61	0.105	0.12
*ID3*	− 0.073	0.28	0.040	0.56	− 0.027	0.70	0.061	0.37
*TGIF2*	− 0.049	0.47	− 0.081	0.23	− 0.179	0.01	0.169	0.01
*INHBA*	− 0.057	0.41	− 0.071	0.30	0.169	0.01	− 0.083	0.23
*ACVR1C*	0.051	0.46	− 0.087	0.20	− 0.064	0.36	0.075	0.27
*BMP4*	0.019	0.78	0.004	0.95	0.091	0.19	− 0.174	0.01
*BMP2*	0.071	0.30	0.224	0.001	− 0.033	0.63	0.086	0.21
*ID1*	0.025	0.72	0.017	0.81	− 0.142	0.04	0.084	0.22
*E2F5*	− 0.070	0.31	− 0.191	0.00	0.059	0.39	0.033	0.63
*FST*	− 0.092	0.18	− 0.020	0.77	− 0.034	0.62	0.143	0.04
*AMHR2*	0.041	0.55	0.022	0.75	0.103	0.13	− 0.048	0.48
*MYC*	− 0.085	0.21	− 0.074	0.28	− 0.157	0.02	0.100	0.14
*THBS1*	− 0.009	0.90	− 0.016	0.82	− 0.017	0.80	0.079	0.25
*LEFTY2*	0.013	0.85	− 0.035	0.61	0.120	0.08	− 0.108	0.11
*GDF7*	0.017	0.80	− 0.122	0.07	0.099	0.15	0.024	0.72
*CDKN2B*	0.136	0.04	− 0.027	0.69	0.197	0.00	− 0.061	0.37
*BMP6*	0.022	0.75	0.062	0.36	0.148	0.03	− 0.004	0.96
*GDF6*	− 0.086	0.21	0.072	0.29	0.132	0.05	− 0.042	0.54
*INHBB*	− 0.175	0.01	0.001	0.99	0.017	0.80	− 0.039	0.57
*TGFBR2*	− 0.018	0.79	− 0.196	0.004	− 0.032	0.65	0.041	0.55
*PITX2*	0.122	0.07	− 0.052	0.44	− 0.066	0.34	0.045	0.51
*ID4*	− 0.048	0.48	− 0.064	0.35	0.025	0.71	− 0.086	0.21
*TGIF1*	0.065	0.34	0.057	0.41	− 0.068	0.33	0.029	0.68
*TFDP1*	− 0.050	0.47	0.116	0.09	− 0.129	0.06	− 0.035	0.61
*LEFTY1*	0.110	0.11	0.011	0.88	− 0.022	0.75	− 0.021	0.75

### mRNA and miRNA associations

**Fig. 2:**
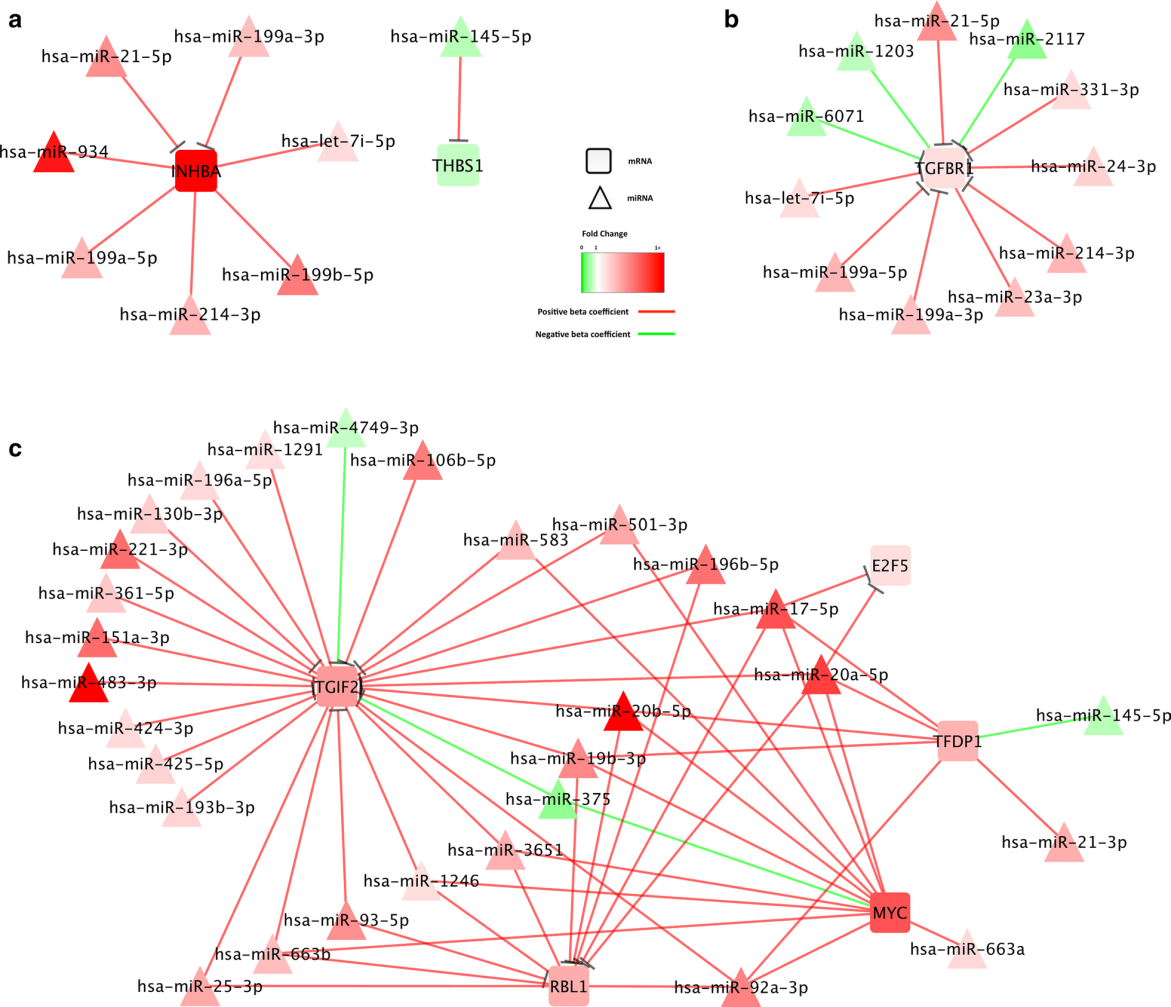
MiRNA-mRNA associations involving genes in the KEGG TGFβ-Signaling Pathway by location of action: extracellular factors (***a***), membrane receptors (***b***), and nuclear factors (***c***). mRNAs are shown as squares and miRNAs are shown in triangles. miRNA-mRNA associations are shown with lines (—) between the miRNA and mRNA pair, with an identified seed match are shown with a (—|) on the mRNA end of the line. Interactions with positive beta coefficients are shown with red lines, and those with negative beta coefficients are shown with green lines. Similarly, upregulated molecules are shown in red, with brighter red indicating a higher FC, and downregulated molecules are shown in green, with brighter green indicating a FC closer to 0

Eleven of the dysregulated genes were associated with miRNA differential expression (Table 4). All of the associated genes were upregulated except for *THBS1*. While two of the genes were only associated with a few miRNAs, *E2F5* with miR-17-5p and miR-20a-5p, and *THBS1* with miR-145-5p, the others were associated with seven or more miRNAs. The miRNAs associated with *E2F5* had seed-region matches with this gene. *RBL1* was associated with 11 miRNAs (five with a seed-region match), *TGFBR1* with 11 miRNAs (nine with a seed-region match), *TGIF2* with 26 miRNAs (eight with a seed-region match), *INHBA* with seven miRNAs (two with a seed-region match), *MYC* with 12 miRNAs, and *TFDP1* with seven miRNAs. Of the miRNAs and mRNAs with a seed-region match four had inverse associations as indicated by a negative beta coefficient (*TGFBR1* with miR-2117 and miR-6071; *TGIF2* with miR-375 and miR-4749-3p). The FC of the differentially expressed miRNAs varied less in the population than the differential expression of the mRNAs. Although variability in the population still existed, when a population FC of > 1.50 or < 0.67 was observed for the miRNA, a much larger percentage of the population had a significant up or downregulated miRNA. The stronger the population FC, the larger percentage of individuals had a similar FC. Figure 1 displays the miRNAs with their associated mRNAs in the KEGG TGFβ-signaling pathway. Figure 2 highlights key components of the pathway, including those genes acting as extracellular factors (Fig. 2a), those acting as membrane receptors (Fig. 2b) and those acting as nuclear factors (Fig. 2c) that were associated miRNAs. Seed region matches are shown with a –| between the miRNA and mRNA, while interactions without seed matches are shown with a line.

**Table 4:** Associations between differentially expressed genes in the TGFβ-signaling pathway and differential miRNA expression

Gene name	Fold change	miRNA	Tumor mean	Tumor SD	T Pct with expression	Normal mean	Normal SD	N Pct with expression	Fold change	(95% CI)	% < 0.67	% > 1.5	Beta	FDR p-value
*RBL1*	2.30	hsa-miR-1246	629.21	296.96	100.00	412.81	121.13	100.00	1.52	(1.42, 1.63)	2.90	47.83	0.30	0.0116
		***hsa-miR-17-5p*** ^a^	61.04	48.49	98.47	16.38	10.13	95.93	3.73	(3.24, 4.22)	3.86	83.57	0.32	0.0116
		hsa-miR-196b-5p	17.89	19.62	76.76	5.53	5.43	71.00	3.24	(2.60, 3.88)	–^b^	–	0.23	0.0407
		***hsa-miR-19b-3p***	29.80	23.72	93.13	10.42	9.70	83.84	2.86	(2.46, 3.26)	4.83	72.95	0.23	0.0407
		***hsa-miR-20a-5p***	70.78	59.44	97.83	17.61	12.25	94.56	4.02	(3.47, 4.57)	3.86	83.57	0.32	0.0116
		***hsa-miR-20b-5p***	17.65	15.14	88.59	3.30	3.59	61.07	5.35	(4.36, 6.34)	–	–	0.27	0.025
		hsa-miR-25-3p	30.05	23.13	95.93	12.78	8.12	89.80	2.35	(2.05, 2.65)	4.83	69.57	0.28	0.0181
		hsa-miR-3651	58.66	34.62	99.26	25.92	12.63	99.10	2.26	(2.06, 2.46)	4.35	76.81	0.28	0.0116
		hsa-miR-663b	65.50	24.80	100.00	32.21	14.68	100.00	2.03	(1.87, 2.20)	3.86	79.71	0.25	0.0222
		hsa-miR-92a-3p	121.60	104.09	99.84	41.18	24.92	99.58	2.95	(2.58, 3.33)	3.86	80.19	0.38	0.0116
		***hsa-miR-93-5p***	41.72	32.63	97.78	15.20	9.19	94.61	2.74	(2.39, 3.09)	2.90	77.78	0.28	0.0116
*TGFBR1*	1.52	***hsa-let-7i-5p***	62.16	37.92	98.63	39.97	21.97	98.63	1.56	(1.41, 1.70)	5.31	45.89	0.25	0.031
		hsa-miR-1203	1.76	1.49	43.05	2.83	1.17	64.55	0.62	(0.53, 0.72)	–	–	− 0.23	0.031
		***hsa-miR-199a-3p***	44.83	37.37	95.30	22.53	15.51	94.24	1.99	(1.73, 2.25)	9.66	55.07	0.24	0.031
		***hsa-miR-199a-5p***	20.18	17.86	90.28	9.28	6.76	85.63	2.17	(1.86, 2.49)	7.73	53.14	0.22	0.0458
		***hsa-miR-2117***	1.50	1.98	25.78	4.09	2.08	54.04	0.37	(0.27, 0.46)	–	–	− 0.22	0.0358
		***hsa-miR-214-3p***	13.24	10.86	90.86	6.13	4.14	85.79	2.16	(1.87, 2.45)	6.28	52.17	0.26	0.031
		***hsa-miR-21-5p***	463.11	312.01	99.52	167.37	118.91	99.52	2.77	(2.47, 3.06)	4.35	84.06	0.25	0.031
		hsa-miR-23a-3p	174.68	110.22	99.42	87.53	50.12	99.47	2.00	(1.81, 2.19)	4.35	65.70	0.25	0.031
		***hsa-miR-24-3p***	106.75	61.76	99.63	62.39	29.22	99.74	1.71	(1.57, 1.85)	3.38	56.04	0.24	0.0318
		***hsa-miR-331-3p***	14.64	11.75	95.93	9.30	5.88	93.24	1.57	(1.39, 1.76)	5.31	50.24	0.24	0.031
		***hsa-miR-6071***	0.97	1.07	15.00	1.70	1.11	37.08	0.57	(0.38, 0.76)	–	–	− 0.22	0.0458
*TGIF2*	2.59	***hsa-miR-106b-5p***	15.90	13.81	88.54	5.19	4.13	71.26	3.06	(2.61, 3.51)	–	–	0.23	0.0141
		hsa-miR-1246	629.21	296.96	100.00	412.81	121.13	100.00	1.52	(1.42, 1.63)	2.90	47.83	0.21	0.023
		hsa-miR-1291	5.52	3.41	76.33	3.67	2.12	69.10	1.51	(1.33, 1.68)	–	–	0.20	0.0257
		***hsa-miR-130b-3p***	8.74	4.50	85.68	4.89	3.13	72.05	1.79	(1.60, 1.97)	–	–	0.31	0.0035
		hsa-miR-151a-3p	5.15	3.49	51.56	1.56	1.89	33.81	3.31	(2.59, 4.03)	–	–	0.25	0.0069
		***hsa-miR-17-5p***	61.04	48.49	98.47	16.38	10.13	95.93	3.73	(3.24, 4.22)	3.86	83.57	0.36	0.0035
		hsa-miR-193b-3p	9.12	7.78	86.85	5.42	3.77	83.20	1.68	(1.47, 1.90)	5.80	41.55	0.19	0.0347
		hsa-miR-196a-5p	6.70	7.13	63.71	4.21	3.13	66.93	1.59	(1.31, 1.88)	–	–	0.25	0.0064
		hsa-miR-196b-5p	17.89	19.62	76.76	5.53	5.43	71.00	3.24	(2.60, 3.88)	–	–	0.36	0.0035
		hsa-miR-19b-3p	29.80	23.72	93.13	10.42	9.70	83.84	2.86	(2.46, 3.26)	4.83	72.95	0.30	0.0035
		***hsa-miR-20a-5p***	70.78	59.44	97.83	17.61	12.25	94.56	4.02	(3.47, 4.57)	3.86	83.57	0.34	0.0035
		hsa-miR-20b-5p	17.65	15.14	88.59	3.30	3.59	61.07	5.35	(4.36, 6.34)	–	–	0.38	0.0035
		hsa-miR-221-3p	13.53	12.32	85.42	4.12	4.07	63.71	3.28	(2.73, 3.84)	–	–	0.21	0.0214
		hsa-miR-25-3p	30.05	23.13	95.93	12.78	8.12	89.80	2.35	(2.05, 2.65)	4.83	69.57	0.31	0.0035
		hsa-miR-361-5p	11.62	9.05	88.59	6.20	3.98	81.56	1.87	(1.64, 2.11)	5.31	50.72	0.32	0.0035
		hsa-miR-3651	58.66	34.62	99.26	25.92	12.63	99.10	2.26	(2.06, 2.46)	4.35	76.81	0.25	0.0086
		***hsa-miR-375***	20.50	26.15	82.62	54.53	35.84	99.42	0.38	(0.30, 0.45)	66.18	10.14	− 0.22	0.0192
		hsa-miR-424-3p	39.81	20.91	99.89	25.37	8.09	99.95	1.57	(1.45, 1.69)	3.86	47.83	0.22	0.0167
		hsa-miR-425-5p	11.76	8.74	83.62	6.97	4.39	80.35	1.69	(1.51, 1.87)	6.76	47.83	0.19	0.0299
		***hsa-miR-4749-3p***	8.01	4.10	95.88	12.04	3.97	99.05	0.67	(0.61, 0.72)	42.51	4.83	− 0.22	0.0148
		***hsa-miR-483-3p***	9.73	28.36	43.21	1.88	2.09	48.97	5.17	(2.09, 8.25)	–	–	0.22	0.0124
		hsa-miR-501-3p	7.07	3.42	95.99	2.95	1.65	84.79	2.39	(2.15, 2.64)	4.35	69.08	0.29	0.0035
		hsa-miR-583	6.61	3.92	87.43	3.22	3.20	71.95	2.05	(1.73, 2.37)	–	–	0.18	0.046
		hsa-miR-663b	65.50	24.80	100.00	32.21	14.68	100.00	2.03	(1.87, 2.20)	3.86	79.71	0.25	0.0051
		hsa-miR-92a-3p	121.60	104.09	99.84	41.18	24.92	99.58	2.95	(2.58, 3.33)	3.86	80.19	0.38	0.0035
		***hsa-miR-93-5p***	41.72	32.63	97.78	15.20	9.19	94.61	2.74	(2.39, 3.09)	2.90	77.78	0.27	0.0051
*INHBA*	9.34	hsa-let-7i-5p	62.16	37.92	98.63	39.97	21.97	98.63	1.56	(1.41, 1.70)	5.31	45.89	0.26	0.0452
		hsa-miR-199a-3p	44.83	37.37	95.30	22.53	15.51	94.24	1.99	(1.73, 2.25)	9.66	55.07	0.32	0.0163
		***hsa-miR-199a-5p***	20.18	17.86	90.28	9.28	6.76	85.63	2.17	(1.86, 2.49)	7.73	53.14	0.31	0.0203
		hsa-miR-199b-5p	4.69	4.05	39.41	1.53	1.67	32.54	3.07	(2.28, 3.87)	–	–	0.29	0.0163
		hsa-miR-214-3p	13.24	10.86	90.86	6.13	4.14	85.79	2.16	(1.87, 2.45)	6.28	52.17	0.34	0.0163
		***hsa-miR-21-5p***	463.11	312.01	99.52	167.37	118.91	99.52	2.77	(2.47, 3.06)	4.35	84.06	0.28	0.0163
		hsa-miR-934	4.36	3.61	82.99	0.94	1.36	45.59	4.66	(3.44, 5.88)	–	–	0.40	0.0163
*E2F5*	1.52	***hsa-miR-17-5p***	61.04	48.49	98.47	16.38	10.13	95.93	3.73	(3.24, 4.22)	3.86	83.57	0.30	0.0407
		***hsa-miR-20a-5p***	70.78	59.44	97.83	17.61	12.25	94.56	4.02	(3.47, 4.57)	3.86	83.57	0.31	0.0407
*MYC*	3.70	hsa-miR-1246	629.21	296.96	100.00	412.81	121.13	100.00	1.52	(1.42, 1.63)	2.90	47.83	0.27	0.0163
		hsa-miR-17-5p	61.04	48.49	98.47	16.38	10.13	95.93	3.73	(3.24, 4.22)	3.86	83.57	0.35	0.0136
		hsa-miR-19b-3p	29.80	23.72	93.13	10.42	9.70	83.84	2.86	(2.46, 3.26)	4.83	72.95	0.27	0.0163
		hsa-miR-20a-5p	70.78	59.44	97.83	17.61	12.25	94.56	4.02	(3.47, 4.57)	3.86	83.57	0.33	0.0136
		hsa-miR-20b-5p	17.65	15.14	88.59	3.30	3.59	61.07	5.35	(4.36, 6.34)	–	–	0.31	0.0163
		hsa-miR-3651	58.66	34.62	99.26	25.92	12.63	99.10	2.26	(2.06, 2.46)	4.35	76.81	0.28	0.0188
		hsa-miR-375	20.50	26.15	82.62	54.53	35.84	99.42	0.38	(0.30, 0.45)	66.18	10.14	− 0.29	0.0136
		hsa-miR-501-3p	7.07	3.42	95.99	2.95	1.65	84.79	2.39	(2.15, 2.64)	4.35	69.08	0.26	0.0188
		hsa-miR-583	6.61	3.92	87.43	3.22	3.20	71.95	2.05	(1.73, 2.37)	–	–	0.26	0.0233
		hsa-miR-663a	374.83	174.81	100.00	234.91	83.58	100.00	1.60	(1.49, 1.70)	3.86	56.04	0.28	0.0188
		hsa-miR-663b	65.50	24.80	100.00	32.21	14.68	100.00	2.03	(1.87, 2.20)	3.86	79.71	0.33	0.0136
		hsa-miR-92a-3p	121.60	104.09	99.84	41.18	24.92	99.58	2.95	(2.58, 3.33)	3.86	80.19	0.32	0.0136
*THBS1*	0.66	hsa-miR-145-5p	132.97	156.85	99.84	223.14	182.21	100.00	0.60	(0.48, 0.71)	66.18	12.56	0.29	0.0407
*TFDP1*	2.21	hsa-miR-145-5p	132.97	156.85	99.84	223.14	182.21	100.00	0.60	(0.48, 0.71)	66.18	12.56	− 0.25	0.0488
		hsa-miR-17-5p	61.04	48.49	98.47	16.38	10.13	95.93	3.73	(3.24, 4.22)	3.86	83.57	0.33	0.0163
		hsa-miR-19b-3p	29.80	23.72	93.13	10.42	9.70	83.84	2.86	(2.46, 3.26)	4.83	72.95	0.29	0.0233
		hsa-miR-20a-5p	70.78	59.44	97.83	17.61	12.25	94.56	4.02	(3.47, 4.57)	3.86	83.57	0.32	0.0163
		hsa-miR-20b-5p	17.65	15.14	88.59	3.30	3.59	61.07	5.35	(4.36, 6.34)	–	–	0.31	0.0163
		hsa-miR-21-3p	22.68	12.22	96.51	9.89	5.94	87.00	2.29	(2.06, 2.52)	3.38	71.98	0.29	0.0233
		hsa-miR-92a-3p	121.60	104.09	99.84	41.18	24.92	99.58	2.95	(2.58, 3.33)	3.86	80.19	0.34	0.0163

^a^Bolditalics text indicates seed-region match between miRNA and mRNA

^b^Values could not be meaningfully calculated when less than 80% of the population had either a tumor or normal expression value of 0

Several miRNAs were associated with multiple genes (Table 5). Both miR-17-5p and miR-20a-5p were associated with the same three genes (*RPBL1*, *TGIF2*, and *E2F5*) with seed-region matches. MiR-199a-5p and miR-21-5p both had seed region matches to *TGFBR1* and *INHBA*. MiR-93-5p had seed-region matches to both *RBL1* and *TGIF2*. Both miR-1246 and miR-3651 were associated with *RBL1*, *TGIF2*, and *MYC* without seed-region matches. MiR-19b-3p and miR-20b-5p were associated with *TGIF2*, *MYC*, and *TFDP1* without seed-region matches. MiR-501-3p and miR-663a were associated with *MYC*. MiR-92-3p was associated with the most genes without a seed-region match, *RBL1*, *TGIF2*, *MYC*, and *TFDP1*.

**Table 5:** Summary of miRNA and mRNA associations by seed-region match

miRNA	Genes with seed-region match	Genes without seed-region match
hsa-let-7i-5p	*TGFBR1*	*INHBA*
hsa-miR-106b-5p	*TGIF2*	
hsa-miR-1203		*TGFBR1*
hsa-miR-1246		*RBL1, TGIF2, MYC*
hsa-miR-1291		*TGIF2*
hsa-miR-130b-3p	*TGIF2*	
hsa-miR-145-5p	*THBS1*	*TFDP1*
hsa-miR-151a-3p		*TGIF2*
hsa-miR-17-5p	*RBL1, TGIF2, E2F5*	*MYC, TFDP1*
hsa-miR-193b-3p		*TGIF2*
hsa-miR-196a-5p		*TGIF2*
hsa-miR-196b-5p		*RBL1, TGIF2*
hsa-miR-199a-3p	*TGFBR1*	*INHBA*
hsa-miR-199a-5p	*TGFBR1, INHBA*	
hsa-miR-199b-5p		*INHBA*
hsa-miR-19b-3p	*RBL1*	*TGIF2, MYC, TFDP1*
hsa-miR-20a-5p	*RBL1, TGIF2, E2F5*	*MYC, TFDP1*
hsa-miR-20b-5p	*RBL1*	*TGIF2, MYC, TFDP1*
hsa-miR-2117	***TGFBR1*** ^a^	
hsa-miR-21-3p		*TFDP1*
hsa-miR-214-3p	*TGFBR1*	*INHBA*
hsa-miR-21-5p	*TGFBR1, INHBA*	
hsa-miR-221-3p		*TGIF2*
hsa-miR-23a-3p		*TGFBR1*
hsa-miR-24-3p	*TGFBR1*	
hsa-miR-25-3p		*RBL1, TGIF2*
hsa-miR-331-3p	*TGFBR1*	
hsa-miR-361-5p		*TGIF2*
hsa-miR-3651		*RBL1, TGIF2, MYC*
hsa-miR-375	***TGIF2***	*MYC*
hsa-miR-424-3p		*TGIF2*
hsa-miR-425-5p		*TGIF2*
hsa-miR-4749-3p	***TGIF2***	
hsa-miR-483-3p	*TGIF2*	
hsa-miR-501-3p		*TGIF2, MYC*
hsa-miR-583	*TGIF2*	*MYC*
hsa-miR-6071	***TGFBR1***	
hsa-miR-663a		*TGIF2, MYC*
hsa-miR-663b		*RBL1, MYC*
hsa-miR-92a-3p		*RBL1, TGIF2, MYC, TFDP1*
hsa-miR-934		*INHBA*
hsa-miR-93-5p	*RBL1, TGIF2*	

^a^Bolditalics text implies inverse association (negative Beta coefficient) with seed-region match

The combined mRNA:miRNA associations differed somewhat by category of sex, age, disease stage and months of survival (Table 6). While differences in the associations between the association of miRNA and mRNA did not differ for many associations by sex, it is noteworthy that for most of the miRNAs associated with *TGIF2*, correlations were twice as strong among men as among women. Most associations between *RBL1*, *TGIF2*, and *TFDP1* and miRNAs tended to be much stronger for individuals < 55 years of age. Looking at disease stage, more differences in association existed for disease stage II, however several mRNA:miRNA associations were strongest for stage IV. Associations that were strongest for stage IV disease were: *RBL1* with miR-17-5p, miRp19b-3p, miR-20a-5p, miR-25-3p, and miR-3651; *TGFBR1* with miR-1203, miR-2117, mir-21-5p, miR-23a-3p, miR-24-3p, and miR-331-3p; *TGIF2* with miR-106b-5p, miR-1246, miR-1291, miR-130b-3p, miR-17-5p, miR-196a-5p, miR-19b-3p, miR-20a-5p, miR-20b,5p, miR-221-3p, miR-25-3p, miR-361-5p, miR-3651, miR-424-3p, miR-425-5p, miR-4749-3p, miR-501-3p, miR-583, miR-663b, miR-92a-3p, and miR-93-5p; and *TDFP1* with miR-145-5p, miR-17-5p, miR-19b-3p, miR-20a-5p, and miR-20b-5p. Several mRNA:miRNA associations were strongest among those with the fewest survival months including *TGFBR1* with miR-331-3p and miR-21-5p; *TGIF2* with miR-1291, mir-130b-3p, miR-17-5p, miR-196a-5p, miR-19b-3p, miR-20a-5p, miR-20b-5p, miR-221-3p, miR-25-3p, miR-361-5p, miR-424-3p, miR-483-3p, miR-501-3p, miR-583, miR-663b, miR-92a-3p, and miR-93-5p; and *TFDP1* with miR-17-5p, miR-20a-5p, and miR-92a-3p. On the other hand, some mRNA:miRNA associations were strongest among those with better survival, such as *RBL1* with miR-1246, miR-196b-5p, and miR-3651; and *INHBA* with miR-199a-3p, miR-199a-5p, miR-199b-5p, miR-214-3p, and miR-934.

**Table 6:** Spearman correlations between associated mRNA and miRNA by strata of clinical features (sex, age, disease stage, and survival months)

Gene	miRNA	Sex				Age						Disease stage							
		Male		Female		< 55		55–65		> 65		I		II		III		IV	
		(N = 113)	p-value	(N = 94)	p-value	(N = 38)	p-value	(N = 50)	p-value	(N = 119)	p-value	(N = 55)	p-value	(N = 58)	p-value	(N = 69)	p-value	(N = 22)	p-value
*RBL1*	hsa-miR-1246	0.22	0.0179	0.36	0.0004	0.48	0.0024	0.24	0.0972	0.25	0.0064	0.28	0.0359	0.41	0.0013	0.14	0.2587	0.24	0.2797
	***hsa-miR-17-5p*** ^a^	0.35	0.0001	0.32	0.0015	0.60	< 0.0001	0.36	0.0105	0.26	0.0050	0.33	0.0146	0.06	0.6625	0.43	0.0002	0.60	0.0030
	hsa-miR-196b-5p	0.24	0.0112	0.14	0.1709	0.57	0.0002	0.21	0.1450	0.09	0.3364	0.28	0.0356	− 0.02	0.8680	0.32	0.0067	0.07	0.7549
	***hsa-miR-19b-3p***	0.27	0.0043	0.19	0.0714	0.48	0.0024	0.11	0.4631	0.22	0.0163	0.12	0.3670	0.00	0.9856	0.40	0.0007	0.51	0.0151
	***hsa-miR-20a-5p***	0.35	0.0002	0.34	0.0009	0.59	< 0.0001	0.25	0.0785	0.32	0.0005	0.38	0.0040	0.01	0.9170	0.42	0.0003	0.61	0.0024
	***hsa-miR-20b-5p***	0.30	0.0012	0.22	0.0370	0.50	0.0013	0.22	0.1250	0.20	0.0269	0.31	0.0214	0.04	0.7694	0.32	0.0080	0.53	0.0109
	hsa-miR-25-3p	0.34	0.0003	0.29	0.0052	0.62	< 0.0001	0.35	0.0118	0.25	0.0065	0.20	0.1337	0.13	0.3288	0.43	0.0002	0.62	0.0022
	hsa-miR-3651	0.26	0.0048	0.28	0.0063	0.39	0.0150	0.15	0.2986	0.28	0.0018	0.15	0.2693	0.18	0.1725	0.37	0.0016	0.52	0.0136
	hsa-miR-663b	0.25	0.0069	0.32	0.0014	0.20	0.2373	0.17	0.2269	0.33	0.0003	0.46	0.0004	0.16	0.2227	0.29	0.0146	0.26	0.2484
	hsa-miR-92a-3p	0.39	< 0.0001	0.49	< 0.0001	0.50	0.0013	0.34	0.0151	0.44	< 0.0001	0.47	0.0003	0.10	0.4560	0.52	< 0.0001	0.63	0.0016
	***hsa-miR-93-5p***	0.30	0.0013	0.21	0.0398	0.61	< 0.0001	0.34	0.0161	0.12	0.2076	0.13	0.3422	0.13	0.3188	0.29	0.0153	0.53	0.0105
*TGFBR1*	***hsa-let-7i-5p***	0.27	0.0037	0.26	0.0129	0.14	0.4110	0.38	0.0066	0.25	0.0068	0.10	0.4666	0.33	0.0118	0.32	0.0079	0.16	0.4775
	hsa-miR-1203	− 0.21	0.0281	− 0.24	0.0189	− 0.20	0.2245	− 0.17	0.2461	− 0.27	0.0029	− 0.02	0.9031	− 0.26	0.0453	− 0.23	0.0626	− 0.39	0.0713
	***hsa-miR-199a-3p***	0.27	0.0042	0.29	0.0050	0.34	0.0387	0.34	0.0147	0.21	0.0195	0.10	0.4583	0.35	0.0066	0.32	0.0080	0.15	0.5030
	***hsa-miR-199a-5p***	0.21	0.0240	0.29	0.0051	0.30	0.0644	0.32	0.0243	0.17	0.0703	0.20	0.1418	0.26	0.0455	0.22	0.0753	0.12	0.5974
	***hsa-miR-2117***	− 0.21	0.0262	− 0.18	0.0882	0.04	0.8019	− 0.24	0.0928	− 0.24	0.0098	− 0.10	0.4680	− 0.29	0.0261	− 0.06	0.5961	− 0.43	0.0449
	***hsa-miR-214-3p***	0.25	0.0065	0.35	0.0005	0.26	0.1148	0.32	0.0223	0.26	0.0042	0.27	0.0469	0.28	0.0311	0.26	0.0331	0.34	0.1171
	***hsa-miR-21-5p***	0.31	0.0007	0.25	0.0165	0.04	0.8139	0.30	0.0317	0.33	0.0003	0.23	0.0906	0.27	0.0393	0.26	0.0317	0.40	0.0665
	hsa-miR-23a-3p	0.23	0.0149	0.32	0.0015	0.32	0.0518	0.26	0.0645	0.25	0.0070	0.22	0.1009	0.30	0.0203	0.18	0.1376	0.43	0.0441
	***hsa-miR-24-3p***	0.28	0.0030	0.27	0.0080	0.24	0.1525	0.34	0.0164	0.26	0.0043	0.26	0.0524	0.32	0.0154	0.20	0.0953	0.42	0.0533
	***hsa-miR-331-3p***	0.25	0.0066	0.33	0.0013	0.23	0.1605	0.37	0.0086	0.27	0.0027	0.32	0.0179	0.28	0.0334	0.14	0.2477	0.55	0.0078
	***hsa-miR-6071***	− 0.11	0.2430	− 0.15	0.1401	-0.08	0.6403	− 0.07	0.6338	− 0.17	0.0580	− 0.11	0.4264	− 0.18	0.1734	− 0.17	0.1628	0.07	0.7622
*TGIF2*	***hsa-miR-106b-5p***	0.29	0.0018	0.10	0.3172	0.34	0.0374	0.21	0.1478	0.18	0.0526	− 0.01	0.9462	0.15	0.2745	0.20	0.0951	0.59	0.0040
	hsa-miR-1246	0.25	0.0074	0.12	0.2377	0.30	0.0669	0.20	0.1621	0.17	0.0689	0.11	0.4273	0.18	0.1708	0.21	0.0910	0.31	0.1619
	hsa-miR-1291	0.27	0.0035	0.13	0.2298	0.10	0.5614	0.18	0.2210	0.25	0.0058	− 0.02	0.8778	0.28	0.0358	0.11	0.3743	0.41	0.0590
	***hsa-miR-130b-3p***	0.24	0.0109	0.14	0.1648	0.30	0.0669	0.21	0.1521	0.16	0.0766	− 0.13	0.3298	0.25	0.0578	0.20	0.0990	0.73	0.0001
	hsa-miR-151a-3p	0.31	0.0007	0.20	0.0494	0.08	0.6183	0.29	0.0399	0.30	0.0009	0.10	0.4557	0.39	0.0024	0.28	0.0190	0.11	0.6346
	***hsa-miR-17-5p***	0.48	< 0.0001	0.37	0.0003	0.56	0.0002	0.36	0.0094	0.41	< 0.0001	0.26	0.0523	0.36	0.0061	0.40	0.0006	0.72	0.0002
	hsa-miR-193b-3p	0.27	0.0034	0.10	0.3611	0.08	0.6150	0.07	0.6147	0.26	0.0048	0.05	0.7103	0.37	0.0039	0.19	0.1218	0.04	0.8477
	hsa-miR-196a-5p	0.21	0.0254	0.18	0.0841	0.37	0.0208	0.14	0.3489	0.19	0.0423	0.08	0.5775	0.15	0.2700	0.16	0.1804	0.33	0.1328
	hsa-miR-196b-5p	0.43	< 0.0001	0.25	0.0154	0.56	0.0003	0.21	0.1349	0.30	0.0010	0.30	0.0250	0.11	0.4039	0.53	< 0.0001	0.26	0.2357
	hsa-miR-19b-3p	0.35	0.0001	0.25	0.0132	0.47	0.0030	0.26	0.0666	0.26	0.0036	0.17	0.2092	0.20	0.1263	0.34	0.0044	0.53	0.0120
	***hsa-miR-20a-5p***	0.45	< 0.0001	0.37	0.0003	0.56	0.0002	0.32	0.0254	0.41	< 0.0001	0.28	0.0360	0.32	0.0136	0.39	0.0010	0.63	0.0015
	hsa-miR-20b-5p	0.48	< 0.0001	0.20	0.0589	0.48	0.0021	0.29	0.0425	0.35	0.0001	0.25	0.0675	0.15	0.2772	0.41	0.0004	0.74	< 0.0001
	hsa-miR-221-3p	0.32	0.0005	0.00	0.9620	0.38	0.0202	− 0.11	0.4459	0.20	0.0262	− 0.04	0.7897	0.16	0.2220	0.13	0.2938	0.53	0.0111
	hsa-miR-25-3p	0.33	0.0003	0.22	0.0327	0.28	0.0921	0.26	0.0665	0.30	0.0009	0.11	0.4334	0.25	0.0632	0.33	0.0052	0.50	0.0182
	hsa-miR-361-5p	0.43	< 0.0001	− 0.03	0.7573	0.07	0.6766	− 0.01	0.9491	0.32	0.0004	− 0.06	0.6557	0.24	0.0742	0.29	0.0175	0.37	0.0902
	hsa-miR-3651	0.32	0.0006	0.21	0.0377	0.25	0.1276	0.11	0.4537	0.33	0.0002	0.07	0.6324	0.32	0.0137	0.27	0.0275	0.44	0.0417
	***hsa-miR-375***	− 0.24	0.0097	− 0.37	0.0002	− 0.25	0.1368	− 0.21	0.1422	− 0.35	< 0.0001	− 0.43	0.0010	− 0.28	0.0331	− 0.32	0.0081	0.10	0.6545
	hsa-miR-424-3p	0.48	< 0.0001	0.12	0.2327	0.29	0.0823	0.16	0.2742	0.37	< 0.0001	0.08	0.5495	0.31	0.0174	0.34	0.0048	0.74	< 0.0001
	hsa-miR-425-5p	0.22	0.0203	0.07	0.5074	0.12	0.4893	0.06	0.6913	0.19	0.0358	− 0.11	0.4253	0.24	0.0657	0.03	0.8003	0.42	0.0499
	***hsa-miR-4749-3p***	− 0.36	0.0001	− 0.34	0.0008	− 0.45	0.0044	− 0.17	0.2269	− 0.37	< 0.0001	− 0.26	0.0574	− 0.40	0.0021	− 0.24	0.0427	− 0.63	0.0018
	***hsa-miR-483-3p***	0.15	0.1210	0.18	0.0793	0.34	0.0341	0.07	0.6130	0.08	0.3592	0.08	0.5847	0.12	0.3686	0.26	0.0310	0.25	0.2672
	hsa-miR-501-3p	0.31	0.0009	0.11	0.2771	0.26	0.1095	0.09	0.5358	0.26	0.0041	0.08	0.5735	0.25	0.0558	0.19	0.1119	0.46	0.0326
	hsa-miR-583	0.16	0.0848	0.10	0.3551	0.20	0.2319	0.02	0.8934	0.16	0.0911	0.04	0.7836	0.06	0.6521	0.22	0.0653	0.44	0.0399
	hsa-miR-663b	0.43	< 0.0001	0.21	0.0466	0.31	0.0555	0.10	0.4996	0.39	< 0.0001	0.34	0.0120	0.25	0.0618	0.34	0.0048	0.52	0.0138
	hsa-miR-92a-3p	0.53	< 0.0001	0.38	0.0001	0.55	0.0004	0.40	0.0039	0.45	< 0.0001	0.37	0.0053	0.31	0.0170	0.51	< 0.0001	0.70	0.0003
	***hsa-miR-93-5p***	0.33	0.0004	0.20	0.0561	0.36	0.0244	0.21	0.1354	0.26	0.0046	0.00	0.9842	0.27	0.0414	0.26	0.0322	0.72	0.0001
*INHBA*	hsa-let-7i-5p	0.26	0.0058	0.36	0.0004	0.31	0.0573	0.40	0.0039	0.25	0.0059	0.20	0.1439	0.27	0.0377	0.38	0.0011	0.15	0.5063
	***hsa-miR-199a-3p***	0.37	< 0.0001	0.46	< 0.0001	0.43	0.0070	0.49	0.0003	0.38	< 0.0001	0.32	0.0187	0.46	0.0002	0.49	< 0.0001	0.17	0.4619
	hsa-miR-199a-5p	0.39	< 0.0001	0.40	< 0.0001	0.42	0.0082	0.50	0.0002	0.35	0.0001	0.30	0.0279	0.40	0.0017	0.44	0.0002	0.21	0.3442
	hsa-miR-199b-5p	0.35	0.0002	0.32	0.0015	0.37	0.0230	0.55	< 0.0001	0.24	0.0090	0.36	0.0066	0.51	< 0.0001	0.23	0.0537	0.08	0.7306
	hsa-miR-214-3p	0.42	< 0.0001	0.41	< 0.0001	0.49	0.0018	0.42	0.0023	0.37	< 0.0001	0.24	0.0764	0.43	0.0008	0.47	< 0.0001	0.44	0.0383
	***hsa-miR-21-5p***	0.29	0.0017	0.26	0.0111	0.22	0.1852	0.38	0.0059	0.27	0.0030	0.14	0.3099	0.22	0.0928	0.38	0.0011	0.40	0.0640
	hsa-miR-934	0.42	< 0.0001	0.46	< 0.0001	0.36	0.0262	0.45	0.0010	0.46	< 0.0001	0.43	0.0011	0.51	< 0.0001	0.28	0.0195	0.65	0.0012
*E2F5*	***hsa-miR-17-5p***	0.27	0.0033	0.35	0.0005	0.45	0.0051	0.46	0.0009	0.23	0.0103	0.33	0.0142	0.38	0.0031	0.22	0.0636	0.27	0.2295
	***hsa-miR-20a-5p***	0.23	0.0135	0.37	0.0003	0.45	0.0048	0.37	0.0081	0.23	0.0107	0.32	0.0158	0.35	0.0063	0.20	0.1013	0.31	0.1556
*MYC*	hsa-miR-1246	0.29	0.0019	0.17	0.0972	0.48	0.0025	0.09	0.5280	0.23	0.0127	0.22	0.0996	0.21	0.1154	0.21	0.0880	0.39	0.0763
	hsa-miR-17-5p	0.42	< 0.0001	0.43	< 0.0001	0.55	0.0003	0.22	0.1231	0.46	< 0.0001	0.30	0.0250	0.31	0.0189	0.45	0.0001	0.68	0.0005
	hsa-miR-19b-3p	0.29	0.0019	0.29	0.0048	0.42	0.0091	0.10	0.4688	0.33	0.0002	0.09	0.4995	0.22	0.0989	0.38	0.0013	0.63	0.0017
	hsa-miR-20a-5p	0.35	0.0002	0.39	0.0001	0.50	0.0012	0.15	0.2955	0.42	< 0.0001	0.24	0.0761	0.27	0.0410	0.37	0.0017	0.71	0.0002
	hsa-miR-20b-5p	0.33	0.0003	0.24	0.0179	0.46	0.0038	0.07	0.6404	0.34	0.0002	0.03	0.8454	0.21	0.1078	0.44	0.0002	0.59	0.0038
	hsa-miR-3651	0.37	< 0.0001	0.31	0.0026	0.31	0.0597	0.06	0.6713	0.44	< 0.0001	0.30	0.0266	0.22	0.0994	0.30	0.0127	0.70	0.0003
	hsa-miR-375	− 0.35	0.0001	− 0.30	0.0038	− 0.13	0.4324	− 0.31	0.0260	− 0.38	< 0.0001	− 0.27	0.0473	− 0.25	0.0604	− 0.50	< 0.0001	− 0.03	0.8791
	hsa-miR-501-3p	0.26	0.0049	0.16	0.1278	0.39	0.0154	0.13	0.3802	0.23	0.0106	− 0.01	0.9491	0.23	0.0803	0.25	0.0410	0.45	0.0372
	hsa-miR-583	0.30	0.0015	0.12	0.2596	0.37	0.0215	0.11	0.4272	0.18	0.0510	0.08	0.5845	0.29	0.0261	0.23	0.0575	0.37	0.0902
	hsa-miR-663a	0.37	< 0.0001	0.20	0.0482	0.29	0.0734	0.11	0.4509	0.36	< 0.0001	0.31	0.0193	0.25	0.0576	0.33	0.0050	0.26	0.2484
	hsa-miR-663b	0.44	< 0.0001	0.27	0.0074	0.26	0.1196	0.02	0.9154	0.49	< 0.0001	0.35	0.0083	0.29	0.0258	0.38	0.0013	0.48	0.0229
	hsa-miR-92a-3p	0.39	< 0.0001	0.35	0.0006	0.48	0.0023	0.08	0.5984	0.45	< 0.0001	0.29	0.0298	0.21	0.1149	0.40	0.0008	0.65	0.0010
*THBS1*	hsa-miR-145-5p	0.33	0.0003	0.24	0.0192	0.48	0.0020	− 0.02	0.9112	0.31	0.0007	0.01	0.9471	0.49	0.0001	0.35	0.0033	0.38	0.0782
*TFDP1*	hsa-miR-145-5p	− 0.13	0.1771	− 0.34	0.0007	− 0.37	0.0205	− 0.06	0.6888	− 0.25	0.0067	− 0.01	0.9333	− 0.36	0.0056	− 0.15	0.2204	− 0.54	0.0096
	hsa-miR-17-5p	0.42	< 0.0001	0.40	< 0.0001	0.60	< 0.0001	0.39	0.0054	0.36	< 0.0001	0.36	0.0068	0.38	0.0037	0.39	0.0010	0.76	< 0.0001
	hsa-miR-19b-3p	0.36	< 0.0001	0.31	0.0027	0.46	0.0038	0.32	0.0228	0.30	0.0008	0.19	0.1580	0.41	0.0014	0.36	0.0023	0.53	0.0105
	hsa-miR-20a-5p	0.45	< 0.0001	0.37	0.0002	0.57	0.0002	0.40	0.0044	0.36	< 0.0001	0.36	0.0067	0.40	0.0021	0.37	0.0019	0.75	< 0.0001
	hsa-miR-20b-5p	0.36	< 0.0001	0.32	0.0018	0.50	0.0012	0.32	0.0227	0.29	0.0013	0.36	0.0077	0.34	0.0090	0.27	0.0270	0.70	0.0003
	hsa-miR-21-3p	0.29	0.0015	0.26	0.0109	0.36	0.0243	0.08	0.5846	0.31	0.0006	0.20	0.1413	0.29	0.0246	0.32	0.0083	0.22	0.3260
	hsa-miR-92a-3p	0.50	< 0.0001	0.35	0.0005	0.63	< 0.0001	0.42	0.0024	0.38	< 0.0001	0.41	0.0018	0.29	0.0249	0.51	< 0.0001	0.66	0.0008

^a^Bolditalics text indicates seed-region match between miRNA and mRNA

## Discussion

The TGFβ pathway is one of the most important pathways in the development of CRC [1]. In normal cells the TGFβ pathway plays an important role in suppressing growth and tumorigenesis; however, as cancer progresses the TGFβ pathway promotes epithelial-mesenchymal transition, invasion and metastasis [24]. In this study, of the 27 dysregulated genes in this pathway, 13 genes were significantly downregulated. The genes that were downregulated included *BMP5, BMP6* and *BMP2*, in addition to *GDF7* and *GDF6*. We have previously shown an association between *BMP2* SNPs and CRC risk [46]. *BMP5* and *BMP6* play an important role in various cancers; both have been shown to induce apoptosis [47]. *BMP6* has previously been shown to be downregulated in metastatic breast cancer [48]. Our data suggests these genes play an important inhibitory role in CRC tumorigenesis as well, and therefore, are downregulated similar to other cancers.

Growth differentiation factors are also known to have inhibitory effects on growth in human cancers, and both *GDF9a* and *GDF9b* have been associated with breast cancer [47]. We found that both *GDF6* and *GDF7* were downregulated in CRC tissues. While we could find no earlier documentation of these genes having an association with CRC risk, *GDF7* is a ligand in the BMP pathway that has been shown to regulate the Hedgehog and Wnt pathways and has been associated with the pro-inflammatory phenotypes in many diseases including Barrett’s Esophagus [49] and esophageal adenocarcinoma [50]. Additionally, the role of Wnt in CRC has been established [51].

The genes that were upregulated in our study included *BMP4, BMP7, TGFBR1, TGFB2, TGIF1, TGIF2*, and *TFDP1*. *BMP4* is known to regulate the SMAD 1/5 signal transduction pathway and plays an important role in angiogenesis [10]. Our data suggest that in CRC, multiple BMPs have altered expression levels. However, it is interesting to note that downregulated *BMP5*, *BMP6*, and *BMP2* were some of the most consistently downregulated genes in the population, with approximately 80% of cases having expression of these genes downregulated in tumors; *BMP4* and *BMP7* were upregulated in over 50% of CRC. *BMPs* are members of a pleiotropic family of growth factors whose signaling has been shown to protect against colonic polyposis with known mutations that can contribute to the development of the tumor microenvironment [52]. Loss of BMP signals has been cited as one of the two main genetic alterations leading to CRC; disrupted BMP signaling allows tumor growth and expansion [53]. BMPs have been shown to upregulate various cytokines important in cancer development and metastasis, and BMP expression levels have been associated with survival and prognosis in various cancers [54, 55]. Our data support these earlier findings of the importance of BMPs in the development and progression of CRC, in that *BMP7*, *BMP5*, *BMP4*, *BMP2*, and *BMP6* were all significantly differentially expressed in CRC tumors and *BMP4* differential expression was inversely associated with survival months. Moreover, they suggest that BMPs play very diverse roles in regulating the TGFβ pathway. Meaning that through their diverse group of downstream ligands and targets, some members of the BMP family can play an important inhibitory effect in tumorigenesis, and therefore are downregulated in CRC, while others play a stimulatory effect in tumorigenesis, and therefore must be upregulated in CRC.

In addition to evaluating the role of the TGFβ pathway in overall CRC risk, we evaluated gene expression levels for associations with mismatch repair proficiency and deficiency. Our data suggest that several genes in this pathway have significant associations with these phenotypes. Notably, *THBS1* was associated with MSI-specific tumors. Members of the thrombospondin family previously have been associated with differential expression in different CRC phenotypes; *THBS4* has been associated with CIMP high tumors, of which a subset are mismatch repair deficient [56]. *THBS1* has previously been associated with the development of the tumor micro-environment and angiogenesis [57] and metastasis [15]. *AMH* was upregulated in all tumors, but had lower expression levels in MSS tumors and the highest expression levels in MSI tumors. *AMH* is selectively expressed in epithelial cells versus mesenchymal cells and a loss of *AMH* is known to induce EMT in various cancers [58]. AMH signaling induces downstream phosphorylation of *SMAD *1/5/8 [58]. Notably SMADs 1/5/8 also are known to be downstream elements in the JNK and ERK pathways [59]. TGFβ is a known activator of the ERK, JNK and p38 MAPK pathways [60], and our data suggests that *AMH* is a possible mediator of this cross-talk between pathways. We have previously shown that both ERK and JNK dependent pathways are significantly associated with CIMP, MSI and *TP53* phenotypes in colon and rectal cancer [61]. Therefore, it is possible that cross-talk between the TGFβ-signaling pathway and other pro-inflammatory pathways plays an important role in the determination of tumor molecular phenotype.

Our data also showed that eight genes in the TGFβ-signaling pathway are associated with differential miRNA expression. MiRNAs have previously been recognized as important in many pathways that regulate normal versus pathological function, and are dysregulated in most, if not all, cancers [62]. In particular, miRNAs have been suggested as potential modulators of the TGFβ-signaling pathways’ seemingly paradoxical effects in regular colonic tissue and CRC development [24, 63]. Additionally, the canonical BMP pathways have been shown to alter gene expression levels through miRNAs [26]. We have previously shown that miRNAs are extensively dysregulated in CRC [64] and that miRNAs can be used to differentiate between normal tissue and colonic carcinoma and rectal carcinoma at a molecular level [41].

In this study, we show that miRNAs play a role in the TGFβ’s function in CRC development, supporting earlier findings. Most earlier studies have focused on one, or several miRNAs such as miR-21 and miR-155 [65], miR-590 [28], miR-17-92 [29] and miR-494 and miR-126-5p [26] or else focus on select genes in the TGFβ pathway, such as *THBS1* [15]. Here we assessed the associations between every gene in the KEGG TGFβ-signaling pathway and the expression levels of over 800 miRNAs. This allowed us to confirm earlier findings of associations with the miR-17-92 cluster (miR-17, 19, 20 and 92), in addition to detecting previously un-reported associations between a diverse array of miRNAs and genes in the TGFβ-signaling pathway. Moreover, we had previously reported that miR-17-5p, miR-20a-5p, miR-145-5p, miR-3651 and miR-92-3p were associated with both colon and rectal cancer, while miR-4749-3p was associated with rectal cancer only and miR-663a was associated with enriched biologic processes in normal colonic mucosa [41]. Here we expand upon our previous findings and suggest that one of the mechanisms by which these miRNAs are associated with colorectal cancer may involve the dysregulation of the TGFβ pathway.

Our results suggest that the component of the TGFβ-signaling pathway most influenced by miRNAs is that part of the pathway that leads to apoptosis and cell cycle control (see Figs. 1, 2c). Dysregulated genes associated with miRNAs were *TGFβR1*, *TGIF2*, *E2F4/E2F5*, *TFDP1*, *RBL1,* and *MYC*. Within these associations it is clear that many genes are associated with several miRNAs and that the same miRNA could influence the expression, either directly or indirectly, of several genes. Notably in this pathway, miR-17-5p, miR-199a-5p, miR-20a-5p, miR-21-5p, and miR-93-5p have seed region matches with multiple genes as well as associations with other genes without a seed-region match, suggesting more of an indirect association. While it is beyond the scope of this paper to further assess these associations in laboratory-based functionality studies, these findings provide information that can be targeted in such studies to validate these findings. Upon validation, further studies to explore potential for targeting therapeutics based on these results can be undertaken.

Variations in clinical features of tumors have been noted by tumor subtype. Guinney and colleagues evaluated four consensus molecular subtypes (CMS), with CMS4 being characterized by activation of TGFβ-signaling [66]. They observed that CMS4 tumors tended to be diagnosed at more advanced disease stages (stage III and IV) and had slightly worse survival. We noted variation within the TGFβ-signaling pathway in regard to both disease stage and survival months. However, the strongest differences by disease stage and survival months were observed when examining the interaction of miRNAs with mRNAs rather than mRNA expression. We believe that these findings may be important in identifying specific markers for further research and as possible treatment or screening modalities

A challenge in studies such as this, is determining which associations are important for follow-up and hold the potential for incorporating observations into clinical practice in the future. This is illustrated in our data where at the population level several genes were upregulated or downregulated, however a large percentage of cases did not demonstrate these characteristics. Two aspects of this observation are worth noting; first, mRNAs that had the greatest fold changes tended to have a greater percentage of the population expressing those changes. This also has implications for determining cutpoints when conducting statistical analysis. We focused our assessment of miRNAs with those genes that were statistically significant and also had a FC of > 1.50 or < 0.67, hoping to identify more biologically important genes. However, our data suggest that at that level fewer individuals expressed changes but those that did had greater changes. Secondly, we also observed less variability in the population for miRNA expression than for mRNA expression, in that miRNAs that were dysregulated tended to be dysregulated in the majority of the population, which was not the case for mRNAs.

This study has several strengths as well as some limitations. First, while our sample size is small, it is one of the largest available samples with paired tumor/normal data. When evaluating miRNA expression with mRNA expression, we could miss important associations since miRNAs have their impact post-transcriptionally. However, much of the current information on miRNA target genes comes from gene expression data, and any association observed may have important biological meaning, but must be acknowledged as being potentially incomplete [67, 68]. Our epidemiological approach should be seen as the first step in identifying associations that can be further examined in targeted laboratory studies to validate the identified associations that can then lead to targeted therapeutics. As such, this work should in part be viewed as a discovery of miRNAs that specifically are associated with genes in the TGFβ-signaling pathway that can be used to spearhead additional research in this field.

## Conclusions

These data provide support for the role of miRNAs in CRC in part through their association with gene regulation in the TGFβ-signaling pathway. Through our comprehensive evaluation of this signaling pathway we have been able to identify genes within the pathway that are most dysregulated in CRC and how dysregulated miRNAs directly and indirectly influence those genes. While validation of study results is needed, these findings provide a basis for further laboratory evaluation and subsequent targeting of key genes and miRNAs.

## Additional file

**Additional file 1. Table S1:** Genes in KEGG TGFBeta-Signaling Pathway. **Table S2**. Differentially expressed mRNA in MSS tumors. **Table S3**. Differentially expressed mRNA in MSI tumors.

## Abbreviations

CRC: colorectal cancer
FDR: false discovery rate
FC: fold change
KPNC: Kaiser Permanente of Northern California
KEGG: Kyoto encyclopedia of genes and genomics
HR: hazard ratio
miRNA: microRNA
MSI: microsatellite instability
MSS: microsatellite stable
QC: quality control
SEER: surveillance epidemiology and end results
TG: target gene
TF: transcription factor
UTR: untranslated region
