# Supplementary material for: The TGFβ-signaling pathway and colorectal cancer: associations between dysregulated genes and miRNAs
Source: J Transl Med. 2018 Jul 9;16:191. doi: 10.1186/s12967-018-1566-8 (PMC6038278; doi:10.1186/s12967-018-1566-8)
Supplement: Supplementary file 1 — Additional file 1. Table S1: Genes in KEGG TGFBeta-Signaling Pathway. Table S2. Differentially expressed mRNA in MSS tumors. Table S3. Differentially expressed mRNA in MSI tumors. [file 12967_2018_1566_MOESM1_ESM.docx]

| Additional file 1: Table S1. Genes in KEGG TGFβ-Signaling Pathway | |
| --- | --- |
| Gene Name | Description |
| *ACVR1* | activin A receptor type 1 [KO:K04675] [EC:2.7.11.30] |
| *ACVR1B* | activin A receptor type 1B [KO:K13567] [EC:2.7.11.30] |
| *ACVR1C* | activin A receptor type 1C [KO:K13568] [EC:2.7.11.30] |
| *ACVR2A* | activin A receptor type 2A [KO:K04670] [EC:2.7.11.30] |
| *ACVR2B* | activin A receptor type 2B [KO:K13596] [EC:2.7.11.30] |
| *AMH* | anti-Mullerian hormone [KO:K04665] |
| *AMHR2* | anti-Mullerian hormone receptor type 2 [KO:K04672] [EC:2.7.11.30] |
| *BAMBI* | BMP and activin membrane bound inhibitor [KO:K10162] |
| *BMP2* | bone morphogenetic protein 2 [KO:K21283] |
| *BMP4* | bone morphogenetic protein 4 [KO:K04662] |
| *BMP5* | bone morphogenetic protein 5 [KO:K04663] |
| *BMP6* | bone morphogenetic protein 6 [KO:K16620] |
| *BMP7* | bone morphogenetic protein 7 [KO:K16621] |
| *BMP8A* | bone morphogenetic protein 8a [KO:K16622] |
| *BMP8B* | bone morphogenetic protein 8b [KO:K16622] |
| *BMPR1A* | bone morphogenetic protein receptor type 1A [KO:K04673] [EC:2.7.11.30] |
| *BMPR1B* | bone morphogenetic protein receptor type 1B [KO:K13578] [EC:2.7.11.30] |
| *BMPR2* | bone morphogenetic protein receptor type 2 [KO:K04671] [EC:2.7.11.30] |
| *CDKN2B* | cyclin dependent kinase inhibitor 2B [KO:K04685] |
| *CHRD* | chordin [KO:K04657] |
| *CREBBP* | CREB binding protein [KO:K04498] [EC:2.3.1.48] |
| *CUL1* | cullin 1 [KO:K03347] |
| *DCN* | decorin [KO:K04660] |
| *E2F4* | E2F transcription factor 4 [KO:K04682] |
| *E2F5* | E2F transcription factor 5 [KO:K04682] |
| *EP300* | E1A binding protein p300 [KO:K04498] [EC:2.3.1.48] |
| *FST* | follistatin [KO:K04661] |
| *GDF5* | growth differentiation factor 5 [KO:K04664] |
| *GDF6* | growth differentiation factor 6 [KO:K20012] |
| *GDF7* | growth differentiation factor 7 [KO:K20013] |
| *ID1* | inhibitor of DNA binding 1, HLH protein [KO:K04680] |
| *ID2* | inhibitor of DNA binding 2, HLH protein [KO:K17693] |
| *ID3* | inhibitor of DNA binding 3, HLH protein [KO:K17694] |
| *ID4* | inhibitor of DNA binding 4, HLH protein [KO:K17695] |
| *IFNG* | interferon gamma [KO:K04687] |
| *INHBA* | inhibin beta A subunit [KO:K04667] |
| *INHBB* | inhibin beta B subunit [KO:K04667] |
| *INHBC* | inhibin beta C subunit [KO:K04667] |
| *INHBE* | inhibin beta E subunit [KO:K04667] |
| *LEFTY1* | left-right determination factor 1 [KO:K04668] |
| *LEFTY2* | left-right determination factor 2 [KO:K04668] |
| *LTBP1* | latent transforming growth factor beta binding protein 1 [KO:K19559] |
| *MAPK1* | mitogen-activated protein kinase 1 [KO:K04371] [EC:2.7.11.24] |
| *MAPK3* | mitogen-activated protein kinase 3 [KO:K04371] [EC:2.7.11.24] |
| *MINOS1-NBL1* | MINOS1-NBL1 readthrough [KO:K19558] |
| *MYC* | MYC proto-oncogene, bHLH transcription factor [KO:K04377] |
| *NBL1* | neuroblastoma 1, DAN family BMP antagonist [KO:K19558] |
| *NODAL* | nodal growth differentiation factor [KO:K04666] |
| *NOG* | noggin [KO:K04658] |
| *PITX2* | paired like homeodomain 2 [KO:K04686] |
| *PPP2CA* | protein phosphatase 2 catalytic subunit alpha [KO:K04382] [EC:3.1.3.16] |
| *PPP2CB* | protein phosphatase 2 catalytic subunit beta [KO:K04382] [EC:3.1.3.16] |
| *PPP2R1A* | protein phosphatase 2 scaffold subunit Aalpha [KO:K03456] |
| *PPP2R1B* | protein phosphatase 2 scaffold subunit Abeta [KO:K03456] |
| *RBL1* | RB transcriptional corepressor like 1 [KO:K04681] |
| *RBX1* | ring-box 1 [KO:K03868] |
| *RHOA* | ras homolog family member A [KO:K04513] |
| *ROCK1* | Rho associated coiled-coil containing protein kinase 1 [KO:K04514] [EC:2.7.11.1] |
| *RPS6KB1* | ribosomal protein S6 kinase B1 [KO:K04688] [EC:2.7.11.1] |
| *RPS6KB2* | ribosomal protein S6 kinase B2 [KO:K04688] [EC:2.7.11.1] |
| *SKP1* | S-phase kinase associated protein 1 [KO:K03094] |
| *SMAD1* | SMAD family member 1 [KO:K04676] |
| *SMAD2* | SMAD family member 2 [KO:K04500] |
| *SMAD3* | SMAD family member 3 [KO:K04500] |
| *SMAD4* | SMAD family member 4 [KO:K04501] |
| *SMAD5* | SMAD family member 5 [KO:K16790] |
| *SMAD6* | SMAD family member 6 [KO:K04677] |
| *SMAD7* | SMAD family member 7 [KO:K19631] |
| *SMAD9* | SMAD family member 9 [KO:K16791] |
| *SMURF1* | SMAD specific E3 ubiquitin protein ligase 1 [KO:K04678] [EC:2.3.2.26] |
| *SMURF2* | SMAD specific E3 ubiquitin protein ligase 2 [KO:K04678] [EC:2.3.2.26] |
| *SP1* | Sp1 transcription factor [KO:K04684] |
| *TFDP1* | transcription factor Dp-1 [KO:K04683] |
| *TGFB1* | transforming growth factor beta 1 [KO:K13375] |
| *TGFB2* | transforming growth factor beta 2 [KO:K13376] |
| *TGFB3* | transforming growth factor beta 3 [KO:K13377] |
| *TGFBR1* | transforming growth factor beta receptor 1 [KO:K04674] [EC:2.7.11.30] |
| *TGFBR2* | transforming growth factor beta receptor 2 [KO:K04388] [EC:2.7.11.30] |
| *TGIF1* | TGFB induced factor homeobox 1 [KO:K19383] |
| *TGIF2* | TGFB induced factor homeobox 2 [KO:K19553] |
| *THBS1* | thrombospondin 1 [KO:K16857] |
| *TNF* | tumor necrosis factor [KO:K03156] |
| *ZFYVE16* | zinc finger FYVE-type containing 16 [KO:K04679] |
| *ZFYVE9* | zinc finger FYVE-type containing 9 [KO:K04679] |

| Additional file 1: Table S2. Differentially expressed mRNA in MSS tumors | | | | | |  |  |  |  |
| --- | --- | --- | --- | --- | --- | --- | --- | --- | --- |
| Gene Name | Tumor Mean | Tumor SD | Normal Mean | Normal SD | Fold Change | (95% CI) | Adjusted P-Value | % FC<0.67 | % FC>1.5 |
| *BMP5* | 3.91 | 0.31 | 22.62 | 1.68 | 0.17 | (0.14, 0.21) | 7.03E-36 | 84.5 | 3.7 |
| *BMP6* | 4.17 | 0.32 | 16.95 | 1.13 | 0.25 | (0.20, 0.30) | 5.73E-31 | 77.5 | 4.3 |
| *IFNG* | 0.64 | 0.10 | 2.43 | 0.33 | 0.26 | (0.18, 0.38) | 1.47E-10 | 48.7 | 1.6 |
| *BMP2* | 19.90 | 1.03 | 66.50 | 3.12 | 0.30 | (0.26, 0.34) | 5.08E-41 | 82.4 | 3.2 |
| *CDKN2B* | 23.04 | 1.22 | 73.95 | 3.61 | 0.31 | (0.27, 0.36) | 5.33E-40 | 80.7 | 4.8 |
| *GDF7* | 1.50 | 0.15 | 3.68 | 0.33 | 0.41 | (0.32, 0.52) | 3.19E-11 | 56.1 | 7.0 |
| *LEFTY2* | 0.83 | 0.18 | 1.89 | 0.40 | 0.44 | (0.31, 0.62) | 9.93E-06 | 34.8 | 8.0 |
| *AMHR2* | 0.11 | 0.04 | 0.25 | 0.09 | 0.45 | (0.24, 0.82) | 1.28E-02 | 18.7 | 2.1 |
| *ACVR1C* | 7.13 | 0.48 | 14.59 | 0.97 | 0.49 | (0.41, 0.59) | 4.84E-12 | 64.7 | 11.2 |
| *LEFTY1* | 36.78 | 3.97 | 69.76 | 6.01 | 0.53 | (0.41, 0.68) | 3.51E-06 | 59.4 | 16.0 |
| *FST* | 1.88 | 0.22 | 3.33 | 0.41 | 0.56 | (0.42, 0.75) | 1.75E-04 | 38.5 | 7.0 |
| *GDF6* | 0.84 | 0.12 | 1.41 | 0.21 | 0.60 | (0.43, 0.85) | 5.10E-03 | 31.6 | 7.0 |
| *MAPK3* | 62.23 | 2.23 | 93.18 | 3.29 | 0.67 | (0.61, 0.73) | 2.16E-16 | 50.8 | 7.0 |
| *THBS1* | 602.48 | 26.10 | 889.92 | 38.47 | 0.68 | (0.62, 0.74) | 5.19E-15 | 49.7 | 9.6 |
| *SMAD4* | 82.35 | 2.33 | 117.11 | 3.32 | 0.70 | (0.66, 0.75) | 3.18E-21 | 42.2 | 4.3 |
| *SMAD1* | 25.90 | 0.92 | 36.75 | 1.31 | 0.70 | (0.64, 0.77) | 6.46E-12 | 48.7 | 7.5 |
| *SMAD9* | 25.31 | 1.58 | 34.18 | 2.02 | 0.74 | (0.64, 0.86) | 1.67E-04 | 50.8 | 16.6 |
| *CHRD* | 6.78 | 0.50 | 9.07 | 0.64 | 0.75 | (0.62, 0.90) | 3.21E-03 | 43.9 | 17.1 |
| *SMAD2* | 149.81 | 4.34 | 191.66 | 5.52 | 0.78 | (0.74, 0.83) | 3.53E-13 | 37.4 | 7.0 |
| *SMAD7* | 32.89 | 1.26 | 42.04 | 1.59 | 0.78 | (0.71, 0.87) | 1.05E-05 | 39.0 | 17.1 |
| *ZFYVE9* | 49.24 | 1.45 | 62.37 | 1.81 | 0.79 | (0.73, 0.85) | 3.35E-08 | 40.1 | 5.3 |
| *ACVR1B* | 96.99 | 2.61 | 115.56 | 3.07 | 0.84 | (0.78, 0.90) | 9.93E-06 | 34.8 | 9.6 |
| *PPP2CB* | 54.70 | 1.76 | 64.75 | 2.05 | 0.84 | (0.78, 0.92) | 1.26E-04 | 37.4 | 13.4 |
| *SMAD3* | 104.97 | 2.84 | 124.23 | 3.30 | 0.85 | (0.79, 0.91) | 7.71E-06 | 29.4 | 9.1 |
| *NBL1* | 183.63 | 5.96 | 217.25 | 6.99 | 0.85 | (0.79, 0.91) | 1.52E-05 | 33.2 | 10.7 |
| *EP300* | 289.16 | 4.25 | 337.06 | 5.00 | 0.86 | (0.83, 0.89) | 3.48E-13 | 18.7 | 2.7 |
| *ID3* | 33.15 | 1.68 | 38.51 | 1.90 | 0.86 | (0.77, 0.97) | 1.53E-02 | 41.2 | 19.3 |
| *RBX1* | 20.09 | 0.73 | 23.14 | 0.89 | 0.87 | (0.79, 0.95) | 3.91E-03 | 31.0 | 15.5 |
| *RPS6KB2* | 35.11 | 0.97 | 40.40 | 1.14 | 0.87 | (0.81, 0.93) | 1.16E-04 | 29.9 | 14.4 |
| *BMP8B* | 21.80 | 0.81 | 24.58 | 0.95 | 0.89 | (0.80, 0.98) | 2.69E-02 | 36.9 | 23.0 |
| *ACVR2A* | 38.55 | 1.53 | 42.10 | 1.68 | 0.92 | (0.83, 1.01) | 7.51E-02 | 26.7 | 16.0 |
| *ROCK1* | 134.36 | 3.77 | 144.34 | 4.08 | 0.93 | (0.88, 0.99) | 2.25E-02 | 21.4 | 13.4 |
| *NODAL* | 2.42 | 0.23 | 2.52 | 0.28 | 0.96 | (0.74, 1.25) | 7.85E-01 | 33.2 | 11.2 |
| *TNF* | 1.94 | 0.20 | 2.00 | 0.22 | 0.97 | (0.72, 1.30) | 8.53E-01 | 25.7 | 10.7 |
| *CREBBP* | 266.24 | 4.31 | 273.55 | 4.49 | 0.97 | (0.94, 1.01) | 1.94E-01 | 7.5 | 5.9 |
| *DCN* | 75.45 | 4.55 | 76.00 | 4.31 | 0.99 | (0.85, 1.15) | 9.24E-01 | 39.0 | 28.3 |
| *MAPK1* | 179.19 | 3.18 | 177.32 | 3.18 | 1.01 | (0.96, 1.06) | 7.06E-01 | 6.4 | 10.2 |
| *TGFB1* | 39.73 | 1.84 | 39.15 | 1.76 | 1.01 | (0.90, 1.14) | 8.21E-01 | 35.3 | 27.8 |
| *ZFYVE16* | 101.90 | 2.99 | 100.08 | 3.00 | 1.02 | (0.95, 1.09) | 6.42E-01 | 15.0 | 20.9 |
| *ID2* | 26.12 | 1.13 | 25.09 | 1.12 | 1.04 | (0.93, 1.16) | 5.33E-01 | 26.7 | 26.2 |
| *INHBE* | 0.81 | 0.11 | 0.76 | 0.11 | 1.06 | (0.75, 1.49) | 7.85E-01 | 25.1 | 5.3 |
| *PPP2R1B* | 80.09 | 2.15 | 75.79 | 2.08 | 1.06 | (0.99, 1.13) | 1.20E-01 | 11.2 | 16.6 |
| *SP1* | 282.32 | 5.00 | 264.84 | 4.78 | 1.07 | (1.02, 1.11) | 5.10E-03 | 5.3 | 9.1 |
| *SMURF1* | 116.78 | 2.54 | 108.26 | 2.39 | 1.08 | (1.02, 1.14) | 1.41E-02 | 10.7 | 22.5 |
| *BMPR1B* | 3.00 | 0.38 | 2.77 | 0.35 | 1.08 | (0.80, 1.46) | 6.56E-01 | 28.3 | 11.2 |
| *PPP2CA* | 97.83 | 2.83 | 87.90 | 2.59 | 1.11 | (1.04, 1.19) | 2.68E-03 | 11.8 | 20.3 |
| *BMPR1A* | 20.98 | 0.80 | 18.77 | 0.75 | 1.12 | (1.00, 1.25) | 5.37E-02 | 19.8 | 27.3 |
| *PPP2R1A* | 144.61 | 3.09 | 128.40 | 2.82 | 1.13 | (1.07, 1.18) | 1.23E-05 | 8.0 | 19.8 |
| *SKP1* | 90.60 | 2.41 | 80.36 | 2.18 | 1.13 | (1.06, 1.20) | 4.86E-04 | 12.3 | 20.3 |
| *TGFB3* | 16.85 | 1.04 | 14.25 | 0.84 | 1.18 | (1.01, 1.39) | 4.82E-02 | 31.0 | 31.0 |
| *TGFBR2* | 207.95 | 5.17 | 175.84 | 4.41 | 1.18 | (1.11, 1.26) | 1.56E-06 | 11.2 | 32.1 |
| *RPS6KB1* | 49.09 | 1.55 | 40.86 | 1.32 | 1.20 | (1.11, 1.30) | 1.65E-05 | 8.6 | 27.3 |
| *ACVR1* | 33.56 | 1.07 | 27.84 | 0.95 | 1.21 | (1.10, 1.32) | 1.30E-04 | 13.9 | 36.9 |
| *ACVR2B* | 15.45 | 0.63 | 12.52 | 0.55 | 1.23 | (1.11, 1.37) | 1.19E-04 | 18.7 | 36.9 |
| *E2F4* | 72.62 | 2.06 | 58.29 | 1.70 | 1.25 | (1.17, 1.33) | 1.61E-10 | 11.2 | 33.2 |
| *BMP8A* | 5.53 | 0.43 | 4.44 | 0.36 | 1.25 | (1.04, 1.50) | 2.52E-02 | 28.3 | 26.7 |
| *ID4* | 14.03 | 0.89 | 11.16 | 0.74 | 1.26 | (1.05, 1.51) | 1.67E-02 | 29.4 | 35.3 |
| *GDF5* | 0.29 | 0.08 | 0.23 | 0.07 | 1.28 | (0.73, 2.27) | 4.37E-01 | 13.9 | 3.7 |
| *SMAD5* | 118.58 | 3.88 | 92.36 | 3.06 | 1.28 | (1.20, 1.37) | 3.19E-11 | 9.6 | 32.6 |
| *BMPR2* | 224.82 | 5.93 | 174.36 | 4.63 | 1.29 | (1.21, 1.37) | 4.44E-13 | 8.0 | 33.7 |
| *ID1* | 61.46 | 4.01 | 47.28 | 2.81 | 1.30 | (1.11, 1.53) | 2.48E-03 | 32.1 | 37.4 |
| *SMAD6* | 21.57 | 1.02 | 16.53 | 0.85 | 1.30 | (1.16, 1.47) | 3.59E-05 | 17.6 | 40.1 |
| *LTBP1* | 82.41 | 3.20 | 62.82 | 2.50 | 1.31 | (1.18, 1.46) | 5.08E-06 | 19.3 | 39.0 |
| *SMURF2* | 50.56 | 1.77 | 37.75 | 1.40 | 1.34 | (1.23, 1.46) | 2.32E-10 | 11.8 | 38.5 |
| *RHOA* | 277.99 | 5.46 | 207.18 | 4.17 | 1.34 | (1.27, 1.41) | 2.08E-21 | 4.3 | 38.5 |
| *CUL1* | 75.17 | 2.17 | 53.39 | 1.60 | 1.41 | (1.31, 1.51) | 1.57E-16 | 6.4 | 40.6 |
| *AMH* | 4.78 | 0.54 | 3.23 | 0.36 | 1.48 | (1.15, 1.90) | 3.50E-03 | 29.9 | 25.7 |
| *TGFBR1* | 103.38 | 3.61 | 67.72 | 2.37 | 1.53 | (1.39, 1.68) | 2.35E-15 | 5.9 | 49.7 |
| *E2F5* | 46.92 | 1.85 | 30.01 | 1.28 | 1.56 | (1.41, 1.74) | 3.34E-14 | 11.8 | 50.8 |
| *TGIF1* | 79.85 | 2.57 | 48.91 | 1.65 | 1.63 | (1.50, 1.78) | 1.67E-22 | 6.4 | 54.0 |
| *BAMBI* | 9.79 | 0.92 | 5.02 | 0.49 | 1.95 | (1.52, 2.50) | 7.45E-07 | 26.7 | 29.9 |
| *INHBB* | 6.29 | 0.65 | 2.98 | 0.31 | 2.11 | (1.64, 2.72) | 7.10E-08 | 22.5 | 31.6 |
| *BMP4* | 50.63 | 3.34 | 23.26 | 1.50 | 2.18 | (1.85, 2.56) | 4.84E-17 | 13.4 | 53.5 |
| *TFDP1* | 118.19 | 3.65 | 52.70 | 1.72 | 2.24 | (2.05, 2.45) | 4.02E-41 | 2.1 | 75.4 |
| *TGFB2* | 9.19 | 0.64 | 4.09 | 0.32 | 2.25 | (1.87, 2.71) | 1.37E-14 | 13.9 | 39.0 |
| *PITX2* | 11.62 | 1.34 | 4.92 | 0.91 | 2.36 | (1.51, 3.70) | 3.50E-04 | 16.6 | 28.9 |
| *RBL1* | 55.19 | 2.25 | 22.86 | 0.99 | 2.41 | (2.17, 2.69) | 7.64E-36 | 7.0 | 71.1 |
| *TGIF2* | 62.50 | 2.47 | 22.36 | 0.95 | 2.79 | (2.49, 3.13) | 1.16E-40 | 4.8 | 74.3 |
| *MYC* | 192.41 | 9.95 | 49.64 | 2.67 | 3.88 | (3.40, 4.42) | 8.64E-48 | 5.3 | 84.0 |
| *BMP7* | 40.71 | 3.54 | 8.93 | 0.81 | 4.56 | (3.62, 5.74) | 4.97E-27 | 13.9 | 56.1 |
| *INHBA* | 124.24 | 8.67 | 11.78 | 0.94 | 10.55 | (8.65, 12.87) | 1.86E-55 | 3.2 | 77.0 |
|  |  |  |  |  |  |  |  |  |  |

| Additional file 1: Table S3. Differentially expressed genes in MSI tumors | | | | | | |  |  |  |
| --- | --- | --- | --- | --- | --- | --- | --- | --- | --- |
| GeneName | Tumor Mean | Tumor SD | Normal Mean | Normal SD | Fold Change | (95% CI) | Adjusted P-Value | % FC<0.67 | % FC>1.5 |
| *GDF5* | 0.05 | 0.06 | 0.25 | 0.37 | 0.18 | (0.03, 1.25) | 1.53E-01 | 17.2 | . |
| *BMP5* | 4.16 | 1.13 | 21.21 | 5.31 | 0.20 | (0.09, 0.42) | 1.23E-03 | 82.8 | 3.4 |
| *BMP6* | 5.13 | 1.03 | 17.62 | 3.07 | 0.29 | (0.18, 0.46) | 1.07E-04 | 75.9 | 3.4 |
| *AMHR2* | 0.24 | 0.18 | 0.77 | 0.65 | 0.32 | (0.05, 1.82) | 2.69E-01 | 17.2 | . |
| *GDF7* | 1.14 | 0.37 | 3.20 | 0.91 | 0.36 | (0.18, 0.69) | 1.46E-02 | 51.7 | 13.8 |
| *ID1* | 14.15 | 2.14 | 37.35 | 5.50 | 0.38 | (0.25, 0.58) | 8.48E-04 | 72.4 | 6.9 |
| *CDKN2B* | 31.17 | 4.33 | 77.67 | 10.59 | 0.40 | (0.27, 0.60) | 7.52E-04 | 75.9 | 13.8 |
| *LEFTY2* | 0.24 | 0.22 | 0.59 | 0.46 | 0.41 | (0.10, 1.60) | 2.69E-01 | 27.6 | 3.4 |
| *GDF6* | 1.36 | 0.66 | 2.80 | 1.26 | 0.49 | (0.13, 1.88) | 3.58E-01 | 24.1 | 3.4 |
| *BMP2* | 26.25 | 3.95 | 52.98 | 7.75 | 0.50 | (0.32, 0.76) | 1.05E-02 | 72.4 | 10.3 |
| *ID3* | 19.62 | 2.40 | 37.74 | 4.52 | 0.52 | (0.37, 0.74) | 3.95E-03 | 58.6 | 13.8 |
| *INHBE* | 1.14 | 0.47 | 2.17 | 0.91 | 0.52 | (0.20, 1.40) | 2.69E-01 | 44.8 | 6.9 |
| *PITX2* | 15.21 | 2.58 | 29.00 | 4.80 | 0.52 | (0.32, 0.85) | 3.71E-02 | 65.5 | 13.8 |
| *NODAL* | 1.93 | 0.50 | 3.36 | 0.86 | 0.57 | (0.27, 1.21) | 2.24E-01 | 48.3 | 10.3 |
| *THBS1* | 480.18 | 50.74 | 830.38 | 84.74 | 0.58 | (0.44, 0.76) | 2.61E-03 | 58.6 | 10.3 |
| *LEFTY1* | 17.45 | 5.47 | 30.12 | 6.87 | 0.58 | (0.26, 1.28) | 2.56E-01 | 72.4 | 13.8 |
| *ACVR1C* | 10.17 | 1.25 | 16.79 | 2.10 | 0.61 | (0.42, 0.87) | 2.87E-02 | 62.1 | 6.9 |
| *TGFBR2* | 116.38 | 7.55 | 192.01 | 12.40 | 0.61 | (0.50, 0.73) | 1.07E-04 | 69.0 | 6.9 |
| *ID4* | 6.88 | 0.87 | 11.07 | 1.42 | 0.62 | (0.43, 0.90) | 4.14E-02 | 48.3 | 13.8 |
| *IFNG* | 1.91 | 0.58 | 2.95 | 0.87 | 0.65 | (0.27, 1.54) | 3.81E-01 | 37.9 | 13.8 |
| *FST* | 1.55 | 0.40 | 2.22 | 0.58 | 0.70 | (0.43, 1.14) | 2.31E-01 | 34.5 | 3.4 |
| *MAPK3* | 64.20 | 6.30 | 89.11 | 8.28 | 0.72 | (0.56, 0.93) | 4.14E-02 | 48.3 | 6.9 |
| *SMAD7* | 25.23 | 2.57 | 34.67 | 3.59 | 0.73 | (0.55, 0.97) | 8.00E-02 | 55.2 | 6.9 |
| *ACVR2A* | 39.81 | 3.44 | 54.40 | 4.76 | 0.73 | (0.57, 0.94) | 4.68E-02 | 41.4 | 17.2 |
| *CHRD* | 5.51 | 0.91 | 7.36 | 1.32 | 0.75 | (0.51, 1.09) | 2.18E-01 | 31.0 | 6.9 |
| *TNF* | 2.01 | 0.64 | 2.65 | 0.83 | 0.76 | (0.37, 1.54) | 4.74E-01 | 31.0 | 6.9 |
| *DCN* | 57.99 | 7.51 | 75.32 | 9.81 | 0.77 | (0.53, 1.12) | 2.56E-01 | 34.5 | 24.1 |
| *ACVR1B* | 75.64 | 6.50 | 95.26 | 7.66 | 0.79 | (0.63, 0.99) | 1.04E-01 | 44.8 | 13.8 |
| *LTBP1* | 43.37 | 6.52 | 52.99 | 7.34 | 0.82 | (0.58, 1.16) | 3.29E-01 | 48.3 | 24.1 |
| *ZFYVE9* | 55.60 | 4.34 | 64.41 | 5.19 | 0.86 | (0.69, 1.09) | 2.79E-01 | 31.0 | 20.7 |
| *SMAD3* | 110.67 | 5.76 | 127.17 | 6.74 | 0.87 | (0.75, 1.01) | 1.29E-01 | 20.7 | 6.9 |
| *NBL1* | 168.55 | 12.73 | 187.98 | 14.44 | 0.90 | (0.75, 1.07) | 2.82E-01 | 34.5 | 10.3 |
| *PPP2CB* | 62.23 | 3.92 | 69.15 | 4.54 | 0.90 | (0.75, 1.08) | 3.34E-01 | 24.1 | 6.9 |
| *CREBBP* | 252.58 | 10.25 | 275.93 | 11.50 | 0.92 | (0.83, 1.01) | 1.53E-01 | 13.8 | 3.4 |
| *BMP8B* | 20.88 | 1.86 | 22.79 | 2.12 | 0.92 | (0.76, 1.11) | 4.09E-01 | 24.1 | 6.9 |
| *SMURF1* | 88.57 | 4.44 | 96.64 | 5.03 | 0.92 | (0.80, 1.05) | 2.69E-01 | 13.8 | 10.3 |
| *SMAD1* | 35.17 | 2.61 | 38.37 | 3.16 | 0.92 | (0.74, 1.14) | 4.68E-01 | 31.0 | 20.7 |
| *BMPR2* | 174.74 | 10.14 | 190.29 | 11.29 | 0.92 | (0.77, 1.09) | 3.81E-01 | 27.6 | 10.3 |
| *EP300* | 328.44 | 10.68 | 356.82 | 12.22 | 0.92 | (0.84, 1.01) | 1.46E-01 | 3.4 | 6.9 |
| *ZFYVE16* | 103.72 | 4.76 | 111.94 | 5.44 | 0.93 | (0.81, 1.05) | 3.20E-01 | 17.2 | 10.3 |
| *RPS6KB2* | 38.34 | 2.41 | 41.32 | 2.77 | 0.93 | (0.77, 1.12) | 4.68E-01 | 20.7 | 13.8 |
| *ACVR1* | 29.81 | 2.47 | 30.86 | 2.73 | 0.97 | (0.75, 1.24) | 7.97E-01 | 27.6 | 34.5 |
| *INHBB* | 3.28 | 0.56 | 3.29 | 0.60 | 1.00 | (0.70, 1.41) | 9.78E-01 | 20.7 | 24.1 |
| *RBX1* | 25.90 | 2.23 | 24.69 | 2.30 | 1.05 | (0.83, 1.33) | 7.24E-01 | 17.2 | 31.0 |
| *TGFB2* | 8.90 | 1.49 | 8.43 | 1.54 | 1.06 | (0.64, 1.75) | 8.38E-01 | 31.0 | 27.6 |
| *PPP2R1A* | 126.03 | 7.63 | 118.15 | 7.49 | 1.07 | (0.93, 1.22) | 4.09E-01 | 10.3 | 13.8 |
| *SMAD9* | 37.72 | 7.03 | 34.93 | 6.26 | 1.08 | (0.67, 1.74) | 7.72E-01 | 41.4 | 37.9 |
| *SMAD4* | 145.67 | 7.52 | 134.00 | 7.30 | 1.09 | (0.93, 1.27) | 3.54E-01 | 10.3 | 13.8 |
| *BMP8A* | 4.16 | 1.02 | 3.82 | 1.00 | 1.09 | (0.69, 1.72) | 7.45E-01 | 31.0 | 17.2 |
| *TGFB3* | 17.21 | 2.36 | 15.77 | 2.23 | 1.09 | (0.74, 1.62) | 7.06E-01 | 34.5 | 34.5 |
| *MAPK1* | 215.16 | 8.11 | 195.12 | 7.76 | 1.10 | (0.99, 1.22) | 1.33E-01 | 6.9 | 10.3 |
| *PPP2R1B* | 86.72 | 3.65 | 78.09 | 3.63 | 1.11 | (0.98, 1.26) | 1.72E-01 | 10.3 | 17.2 |
| *BMPR1A* | 19.18 | 1.65 | 17.18 | 1.63 | 1.12 | (0.88, 1.42) | 4.09E-01 | 31.0 | 20.7 |
| *SKP1* | 97.54 | 4.23 | 86.00 | 4.08 | 1.13 | (0.99, 1.29) | 1.29E-01 | 10.3 | 20.7 |
| *SMAD2* | 240.96 | 11.23 | 211.70 | 10.24 | 1.14 | (0.99, 1.31) | 1.33E-01 | 10.3 | 20.7 |
| *SP1* | 320.96 | 14.79 | 280.66 | 13.21 | 1.14 | (1.02, 1.28) | 6.02E-02 | 6.9 | 20.7 |
| *E2F4* | 68.48 | 3.76 | 59.36 | 3.52 | 1.15 | (1.00, 1.34) | 1.29E-01 | 6.9 | 27.6 |
| *SMAD6* | 18.80 | 2.33 | 15.72 | 2.16 | 1.20 | (0.84, 1.70) | 3.81E-01 | 24.1 | 37.9 |
| *ROCK1* | 211.13 | 13.80 | 173.78 | 11.60 | 1.21 | (1.04, 1.42) | 4.37E-02 | 6.9 | 27.6 |
| *PPP2CA* | 133.03 | 7.71 | 108.76 | 6.71 | 1.22 | (1.04, 1.43) | 4.23E-02 | 3.4 | 31.0 |
| *E2F5* | 45.26 | 3.21 | 36.75 | 2.83 | 1.23 | (1.02, 1.49) | 8.75E-02 | 10.3 | 31.0 |
| *ACVR2B* | 11.60 | 1.50 | 9.29 | 1.35 | 1.25 | (0.93, 1.67) | 2.18E-01 | 31.0 | 34.5 |
| *RPS6KB1* | 53.72 | 2.73 | 42.40 | 2.40 | 1.27 | (1.11, 1.45) | 6.21E-03 | 10.3 | 31.0 |
| *TGFB1* | 55.19 | 5.39 | 43.50 | 4.33 | 1.27 | (0.98, 1.64) | 1.34E-01 | 20.7 | 37.9 |
| *SMURF2* | 52.95 | 3.42 | 40.49 | 2.75 | 1.31 | (1.08, 1.59) | 2.87E-02 | 6.9 | 44.8 |
| *TGIF2* | 34.52 | 2.92 | 25.49 | 2.37 | 1.35 | (1.05, 1.75) | 6.02E-02 | 17.2 | 48.3 |
| *CUL1* | 70.88 | 4.11 | 51.41 | 3.26 | 1.38 | (1.16, 1.64) | 4.54E-03 | 6.9 | 37.9 |
| *BAMBI* | 8.57 | 1.35 | 6.08 | 1.09 | 1.41 | (0.91, 2.18) | 2.08E-01 | 20.7 | 48.3 |
| *RHOA* | 318.03 | 14.05 | 221.67 | 10.35 | 1.43 | (1.27, 1.62) | 2.75E-05 | . | 48.3 |
| *TGIF1* | 89.48 | 7.83 | 61.42 | 5.73 | 1.46 | (1.12, 1.89) | 2.50E-02 | 20.7 | 62.1 |
| *TGFBR1* | 128.74 | 9.22 | 87.37 | 6.56 | 1.47 | (1.21, 1.79) | 2.61E-03 | 10.3 | 55.2 |
| *BMPR1B* | 5.37 | 2.34 | 3.61 | 1.77 | 1.49 | (0.55, 4.04) | 4.68E-01 | 24.1 | 13.8 |
| *ID2* | 29.65 | 4.23 | 19.76 | 2.85 | 1.50 | (1.02, 2.21) | 9.88E-02 | 20.7 | 69.0 |
| *SMAD5* | 164.69 | 8.18 | 105.29 | 5.73 | 1.56 | (1.35, 1.82) | 3.02E-05 | 3.4 | 51.7 |
| *RBL1* | 43.59 | 3.49 | 27.24 | 2.56 | 1.60 | (1.25, 2.05) | 3.48E-03 | 13.8 | 58.6 |
| *BMP7* | 20.19 | 5.22 | 10.58 | 2.87 | 1.91 | (0.89, 4.11) | 1.72E-01 | 34.5 | 17.2 |
| *TFDP1* | 125.81 | 8.19 | 63.28 | 4.46 | 1.99 | (1.64, 2.41) | 5.42E-06 | 10.3 | 82.8 |
| *BMP4* | 37.54 | 5.49 | 17.07 | 2.63 | 2.20 | (1.47, 3.28) | 2.61E-03 | 17.2 | 48.3 |
| *MYC* | 111.44 | 10.82 | 45.58 | 4.70 | 2.44 | (1.83, 3.27) | 2.75E-05 | 10.3 | 69.0 |
| *AMH* | 16.68 | 4.24 | 4.89 | 1.50 | 3.41 | (1.71, 6.81) | 5.67E-03 | 13.8 | 41.4 |
| *INHBA* | 119.39 | 25.52 | 34.22 | 10.78 | 3.49 | (1.51, 8.04) | 1.92E-02 | 13.8 | 62.1 |
